# Chromatin Remodeling Complex NuRD in Neurodevelopment and Neurodevelopmental Disorders

**DOI:** 10.3389/fgene.2019.00682

**Published:** 2019-07-24

**Authors:** Anke Hoffmann, Dietmar Spengler

**Affiliations:** Epigenomics of Early Life, Translational Research in Psychiatry, Max Planck Institute of Psychiatry, Munich, Germany

**Keywords:** nucleosome remodeling and deacetylase, neural stem cell, neural progenitor cell, neurodevelopment, corticogenesis, neurodevelopment disorders, schizophrenia, bipolar disorder

## Abstract

The nucleosome remodeling and deacetylase (NuRD) complex presents one of the major chromatin remodeling complexes in mammalian cells. Here, we discuss current evidence for NuRD’s role as an important epigenetic regulator of gene expression in neural stem cell (NSC) and neural progenitor cell (NPC) fate decisions in brain development. With the formation of the cerebellar and cerebral cortex, NuRD facilitates experience-dependent cerebellar plasticity and regulates additionally cerebral subtype specification and connectivity in postmitotic neurons. Consistent with these properties, genetic variation in NuRD’s subunits emerges as important risk factor in common polygenic forms of neurodevelopmental disorders (NDDs) and neurodevelopment-related psychiatric disorders such as schizophrenia (SCZ) and bipolar disorder (BD). Overall, these findings highlight the critical role of NuRD in chromatin regulation in brain development and in mental health and disease.

## Introduction

Epigenetic mechanisms are key to establish stable yet malleable gene expression during (neuro-) development and beyond (Jaenisch and Bird, 2003; Sweatt, 2013). Perturbations in epigenetic regulation, whether through genetic variation (Murgatroyd and Spengler, 2012; Mastrototaro et al., 2017) or through environmental insults (Zhang and Meaney, 2010; Hoffmann and Spengler, 2014; Hoffmann et al., 2017a), trigger long-lasting changes in gene expression that can contribute to future mental health and disease (Hanson and Gluckman, 2014; Hoffmann et al., 2016; Hoffmann et al., 2017a). Major epigenetic mechanisms consist of covalent DNA modifications (e.g., CpG methylation), posttranslational modifications (PTMs) of core histones, nucleosome positioning, and noncoding RNA (Jaenisch and Bird, 2003; Schübeler, 2015; Yadav et al., 2018). All of these act tightly together in the control of gene expression.

Changes in gene expression take place within the mammalian cell’s 5-µm-sized nucleus, in which the genomic DNA is highly compacted *via* specialized proteins, the so-called histone, to fit the limited space. While “open” (euchromatic) regions are accessible to nuclear factors, “closed” (heterochromatic) regions preclude in general transcriptional activation. However, heterochromatic regions can be quickly modified [e.g., acetylated (Rizzi et al., 2004)] in response to metabolic or environmental stress (e.g., heat shock) to enhance transcription of “defense” genes that prevent the formation and accumulation of toxic protein aggregates.

Factors that remodel the configuration of chromatin control gene transcription programs and frame the response to intrinsic and extrinsic signals with broad implications for cellular state. Nucleosome remodeling and deacetylase (NuRD) presents one of four major ATP (adenosine triphosphate)-dependent chromatin remodeling complexes and has been identified originally as a transcriptional silencer (Ho and Crabtree, 2010). Meanwhile, this view has been revised in light of NuRD’s multifarious effects on gene transcription, including gene activation, in embryonic development, cancer, and aging (Lai and Wade, 2011).

Here, we review major advances on NuRD’s role in neurodevelopment and neurodevelopmental disorders (NDDs). Initially, we define core features of mammalian NuRD in pluripotent stem cell lines as an easy accessible model for basic studies. From there, we move on to the role of NuRD in neural progenitor and cortical cells *in vivo*, with a focus on cell lineage specification, neuronal differentiation, and maturation. Thereafter, we examine current evidence for a role of NuRD in common neurodevelopmental and neurodevelopment-related psychiatric disorders. Concluding, we consider further steps to be taken to corroborate NuRD’s function in mental health and disease and how such knowledge may help to reframe current disease concepts.

### Methods

For the literature selection process, we utilized the databank PubMed and applied combinations of the following search terms: NuRD, chromatin remodel* (e.g., remodeler, remodeling), embryonic* or neural* (e.g., development, stem cell), and neurodevelopmental* or neuropsychiatric* (e.g., disorder, symptom, disease). Later search results were narrowed to intellectual disability (ID), autism spectrum disorders (ASDs), schizophrenia (SCZ), and bipolar disorder (BD). The search process covered the period 1980 to June 2019. Only studies in English that investigated NuRD or its core subunits were included. In addition, we followed up references from the identified publications, of similar articles indicated by PubMed, and of citatory publications by referring to Google Scholar^®^.

### NuRD in Chromatin Remodeling

The nuclear genome of eukaryotic cells is organized into chromatin in which DNA, RNA, and associated proteins are packaged together. Chromatin provides a large source of information that extends from the linear chromatin template to the basic building block, the nucleosome, and from there to three-dimensional (3D) structures. The nucleosome consists of ∼147 base pairs of double-stranded DNA coiled around core histones with chromatin-free DNA segments linking single nucleosomal units (Sitbon et al., 2017). Higher-level chromatin structures arise from further winding of the chromatin template and impose further compaction on the DNA. These 3D structures also contribute to the formation of interacting chromatin loops and of topologically associating domains that impact long-range gene regulation and the expression of functionally related gene groups. Core histones consist of highly conserved alkaline proteins, which serve as substrates for various PTMs. Well-studied PTMs include histone methylation, acetylation, phosphorylation, ubiquitination, and sumoylation. Together, these modifications produce modular signatures influencing chromatin organization (Sitbon et al., 2017). Chromatin remodelers, histone- and DNA-modifying enzymes, RNA, and a vast array of multifarious transcription factors (TFs), jointly read and shape a versatile chromatin landscape. At each structural level, chromatin modulation allows plastic responses to gene regulatory signals (Jaenisch and Bird, 2003) and places chromatin at the center of stable, yet adaptable, gene expression.

A number of mammalian chromatin remodeling complexes consume energy gained from ATP hydrolysis to shift nucleosomes relative to the DNA sequence. This process facilitates chromatin remodeling and TF access to DNA-binding sites (Hota and Bruneau, 2016). ATP-dependent chromatin remodeling complexes consist primarily of a single ATPase that contains a high-affinity substrate binding and a catalytic site. The catalytic activity of the ATPase is regulated through multiple associated subunits that direct additionally complex binding throughout the genome (Hota and Bruneau, 2016).

**Figure 1:**
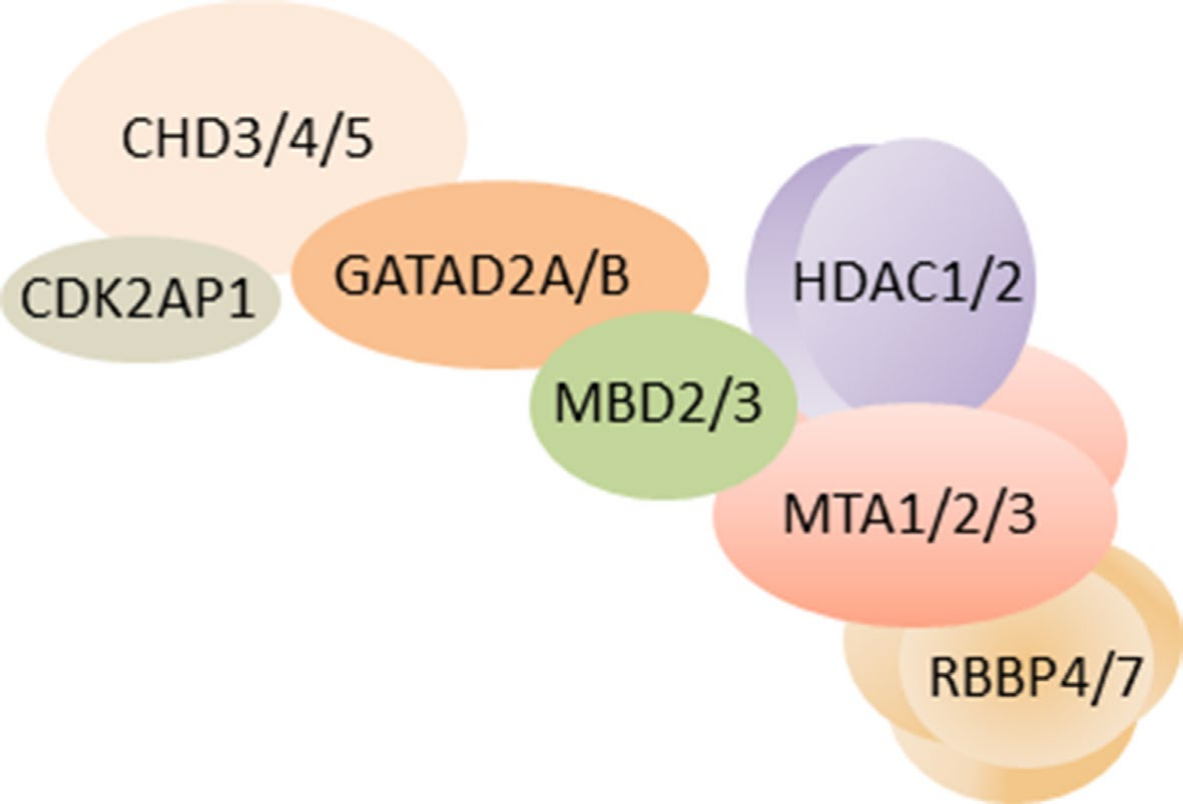
Schematic of NuRD. As of yet, the three-dimensional structure of overall NuRD has not been determined and proteins are depicted at approximate stoichiometry. Mass spectrometry suggests that NuRD consists of seven different proteins: the nucleosome remodeling subcomplex contains one CHD3/4/5 protein, one CDK2AP1 protein, and one GATAD2A/B protein. One MBD2/3 protein bridges the remodeling subcomplex to the histone deacetylase subcomplex, which consist of HDAC1/2 proteins, two MTA1/2/3 proteins, and four RBBP4/7 proteins. Among these subunits, the two paralogs of MBD are found to be mutually exclusive, alike the three paralogs of MTA. It is important to note that other proteins may be associated with NuRD in a tissue-specific and task-orientated manner. Likewise, individual subunits such as CHD may assemble specifically in NuRD across distinct developmental stages, tissues, and cell types and endow NuRD with distinct regulatory properties. Schematic adapted from (Bornelöv et al., 2018), attribution CC BY.

NuRD (*alias* NRD or Mi-2) is one such macromolecular protein complex that is unique in combining chromatin remodeling and protein deacetylase activity (**Figure 1**). The remodeling subcomplex consists of one ATPase (chromodomain helicase DNA-binding protein 3/4/5; CHD3/4/5) that associates with one GATAD2A/B (GATA zinc finger domain containing protein 2A/B, *alias* p66α/β) protein, and the DOC1/CDK2AP1 protein (deleted in oral cancer/CDK2 associated protein 1). The associated deacetylase subcomplex consists of HDAC1/2 (class I lysine deacetylase1/2) proteins, two metastasis tumor antigen proteins (MTA1, MTA2, and/or MTA3), and the histone chaperones RBBP4/7 (retinoblastoma binding protein 4/7). In addition, the zinc finger proteins SALL1/4 (i.e., SAL-like 1/4) have been found to associate with the deacetylase subcomplex in a cell type and tissue-specific manner (Allen et al., 2013; Torchy et al., 2015). Lastly, one of the methyl-CpG-binding domain proteins 2/3 (MBD2/3) bridges the two subcomplexes in entire NuRD.

CHD, GATAD2, MBD, and MTA proteins are defining subunits of NuRD, whose differential assembly modulates its function in a cell-type-specific manner. For example, NuRD containing CHD3 or CHD4 has distinct although overlapping functions (Hoffmeister et al., 2017) and fulfills distinct roles in corticogenesis [see below (Nitarska et al., 2016)]. Likewise, the bridging proteins Mbd2 and Mbd3 are mutually exclusive in NuRD with Mbd3 required for early postimplantation development in mice, while Mbd2 is not [see below (Günther et al., 2013; Wood and Zhou, 2016)]. Mbd2 contains a *bona fide* methyl-CpG-binding domain thought to recruit NuRD to methylated DNA. By contrast, Mbd3’s methyl-CpG-binding like-domain is incapable of high-affinity methylated DNA binding (Zhang et al., 1999) and is dispensable in normal differentiation and development. Accordingly, Mbd3 operates solely to bridge remodeling and deacetylase subcomplexes within entire NuRD (Zhang et al., 2016). Although Mbd2/3 is necessary for entire NuRD, functional and genetic data raise the possibility that the CHD4 subcomplex can also function on its own during first lineage decisions in embryonic development (O’Shaughnessy-Kirwan et al., 2015). It remains to be clarified to what degree this behavior can be generalized to other developmental conditions and whether it applies to the deacetylase subcomplex as well.

NuRD is as an abundant chromatin-associated complex that binds on a genome-wide scale to nearly all active enhancers and promoters in embryonic stem cells (ESCs) (Günther et al., 2013; Miller et al., 2016; Stevens et al., 2017). Current binding data support a global localization model and suggest that NuRD has a general affinity for open chromatin regions associated with transcriptional activity. Furthermore, a broad variety of TFs bound at specific DNA-binding sites interacts with NuRD to modify gene expression (e.g., Aguilera et al., 2011; Liang et al., 2017). Owing to NuRD’s genome-wide presence, such TFs are thought to increase NuRD’s local availability, rather than to recruit it *ab initio*. Intriguing as it is, this hypothesis still needs further experimental validation, e.g., by determining local NuRD availability dependent on transcriptional activation status and/or by kinetic analysis of NuRD/TF interactions.

Taken together, NuRD regulates gene expression by combining chromatin remodeling and protein deacetylase activity. Similar to other chromatin-modifying complexes (Ho and Crabtree, 2010), changes in NuRD’s subunit composition correlates, at least in part, with distinct changes in function to match the needs of specific cell types and developmental stages (see below).

### NuRD in Embryonic Stem Cells and Embryonic Development

ESCs offer a unique resource for the investigation of mitotic self-renewal and differentiation into virtually any cell type in the presence of appropriate signals. ESCs can be kept as fairly homogeneous population in unlimited amounts as opposed to heterogeneous and/or inaccessible tissues from living organism. Thus, ESCs provide a tractable model to gain mechanistic insight into NuRD’s basic functions that can also inform rodent and human studies referred to below.

To assess the role of the closely related Mbd proteins, Hendrich et al. (2001) generated a set of knockout mice: Embryos without *Mbd3*, but not without *Mbd2*, developed fatal postimplantation patterning defects, suggesting a need of Mbd3 for functional NuRD at this early stage. As a more tractable model, Kaji et al. (2006) subsequently established an *Mbd3*-null ESC line: although these cells showed under growth conditions high expression of pluripotency genes, they grew more slowly, yet without signs of spontaneous differentiation, relative to their wild-type counterpart. Importantly, NuRD complexes were no longer formed in *Mbd3*-deficient ESCs supporting Mbd3’s essential role as bridging factor. In contrast to wild-type ESCs, which efficiently formed all three germ layers under differentiation conditions, the differentiation potential of *Mbd3*-null ESCs was constrained, although not completely eliminated. At the same time, expression of *Oct4* and *Nanog*, two genes with a pivotal role in pluripotency, was maintained in mutated ESCs and retained them in a state of self-renewal.

Collectively, this study strengthens the evidence that Mbd3 is required for NuRD formation in ESCs and assigns to NuRD/Mbd3 a role in self-renewal and lineage commitment.

Early studies on NuRD binding in ESCs (Hu and Wade, 2012) indicated a subtle balance between transcriptional activation and inhibition at NuRD-bound genes sharing an essential role in self-renewal and development. With the advent of genome-wide ChIP-seq (chromatin immunoprecipitation sequencing), Baubec et al. (2013) established genome-wide binding profiles for the family of methyl-CpG-binding proteins in ESCs and ESC-derived neuronal cells. *In vivo* binding of MBD2 required a functional MBD domain (absent in MBD3), and the existence of methyl-CpGs was largely proportional to the local methylation density, and thus mapped to inactive regulatory regions. By contrast, when MBD2 was present in a complex with NuRD, it mapped to a subgroup of methylation-free active regulatory sites. Notably, MBD3 likewise occupied these regions independent of canonical DNA methylation. These genomic regions contained a low number of unmethylated CpG residues, but were enriched in methylation and acetylation marks characteristic of active chromatin (histone 3 lysine 4 methylation and histone 3 lysine 27 acetylation). Moreover, these sites were also DNAse I hypersensitive, another indicator of an open chromatin configuration. Interestingly, a high percentage of these MBD3 sites and the subgroup of methylation-free MBD2 sites contained tissue-specific regulatory regions, active promoters, and enhancers.

How can these findings explain NuRD’s role in balancing ESC self-renewal versus differentiation? Part of the answer came from experiments conducted by Reynolds et al. (2012): Under self-renewal conditions, NuRD bound to a subset of pluripotency genes (*Klf4*,* Klf5*, and *Tbx3*) and confined their expression. Transcriptional heterogeneity describes the expression of one gene or a group of genes to varying degrees in a homogeneous cell population. Such variability in gene expression is thought to provide means by which stem cells can sort their progeny either to different lineages or to self-renewal. To test this hypothesis, Reynolds et al. quantified *via* immunofluorescence microscopy the abundance of short-lived proteins (such as Klf4 and Klf5) as a proxy to transcriptional output in single cells. In addition, the coding region of one Zfp42 allele was replaced by a destabilized (i.e., short-lived) GFP protein to measure output from the Zfp42 gene by flow cytometry. In support of the hypothesis, wild-type ESCs contained a mixture of cells with low and high expression of NuRD-regulated pluripotency genes, whereas NuRD-deficient ESCs expressed uniformly high levels of pluripotency genes and were impaired in differentiation. This result points to a role of NuRD in fine tuning gene expression rather than in categorical “on–off” switches. To strengthen this conclusion and as a more direct proxy to transcriptional regulation, single-cell sequencing rather than protein-based approaches remains desirable.

To examine this issue further, Bornelöv et al. (2018) established in ESCs an inducible NuRD system with tight temporal resolution. Induction of NuRD activity led to rapid genome-wide reorganization of nucleosome structure at enhancers and promoters and eviction of some chromatin-bound proteins and of RNA polymerase II from these sites. Shortly afterwards, the same or similar protein complexes rebound most genes that showed accordingly only transient changes in nascent mRNA production. By contrast, a subset of NuRD-bound genes showed sustained increases or decreases in nascent mRNA production that required NuRD’s nucleosome remodeling activity. This subset included various developmental genes that triggered lineage commitment during transition phases. Irrespective of this evidence, *Mbd3*-deficient ESCs still seemed to retain the appropriate differentiation trajectory, although they fail to reach a differentiated state (Kaji et al., 2006). A plausible explanation for this discrepancy is that *Mbd3*-null ESCs still express NuRD/Mbd2 that may replace NuRD/Mbd3 in gene regulation during early, but not during late stages of differentiation. Although Mbd2 and Mbd3 mapped to largely identical sites in undifferentiated ESCs when present in a complex with NuRD, even slight differences may become important to and/or increase during late differentiation.

Similar to MBDs, the presence of three MTA proteins for NuRD raises the question to which degree they differ in function. Although weak differences in terms of chromatin binding and protein interactions were detectable (Burgold et al., 2018), MTA proteins successfully replaced each other in NuRD activity in ESCs. These finding is in contrast to mature cells, where MTA proteins fulfill, at least in part, distinct functions. ESCs harboring knockouts of all three *MTAs* kept viable, but showed impairments in both lineage commitment and differentiation trajectories. This result indicates that NuRD ties together consecutive steps in early development.

Taken together, NuRD regulates genome-wide, gradual changes in gene expression in ESCs. NuRD-related transcriptional heterogeneity facilitates transition states, in which NuRD modulates transcription of developmental genes catalyzing lineage commitment and differentiation trajectory.

### NuRD in Cerebellar Cortex Development

For clearness, we group studies on NuRD’s role in neural development, neuronal differentiation, and maturation by brain region (e.g., cerebellum) and cell type [e.g., neural progenitor cell (NPC)] rather than by the order of their appearance and summarize key points in a tabular format (**Table 1**).

**Table 1:** NuRD’s role in brain development and neuronal plasticity.

Reference	Species	Model	Tissue	Region/cell type	Major technique	Major findings
(Yamada et al., 2014)	r, m	in vivo RNAi, conditional *Chd4* knock-out	cerebellum	cerebellar cortex/granule neurons	RNA-seq/ChIP-seq, WCPC, EM	NuRD supports the development of granule neuron parallel fiber/Purkinje cell synapse by repressing inhibitors of presynaptic connectivity during critical post-natal time windows of plasticity.
(Yang et al., 2016)	m	conditional *Chd4* knock-out, in vivo transfection, behavioral tests	cerebellum	cerebellar cortex	RNA-seq/ChIP-seq, Ca^2+^ imaging, histology	NuRD inhibits expression of active genes by deposition of the histone variant H2A.z. Thereby, NuRD controls deactivation of neuronal-activity dependent gene transcription, reduces neuronal pruning during sensitive periods, and regulates behavioral responses.
(Knock et al., 2015)	m	conditional *Mbd3* knock-out	developing neocortex	apical and basal progenitors	IHC, ChIP, microarray, qRT-PCR	NuRD/Mbd3 sustains appropriate cell lineage choice and differentiation programs by terminating pro-neurogenic transcription in both progenitor cells and neuronal progeny.
(Egan et al., 2013)	m, h	in utero electro-poration	developing neocortex	developing neocortex, ESCs, neuroblastoma	shRNA, IHC, microarray, ChIP-Seq	Chd5 facilitates activation of neuronal gene expression and maintains repression of a small cohort of Polycomb repressed genes during embryonic neocortex development.
(Potts et al., 2011)	r	primary neuronal culture	developing cortex	cortex, post-mitotic neurons	shRNA, IHC, Co-IP, ChiP, microarray	Chd5 regulates neuronal genes and chromatin modifiers in embryonic neurons. NuRD/Chd5 also strongly regulates genes associated with aging and Alzheimer’s disease.
(Nitarska et al., 2016)	m	*chd4* knock-out, in utero electro-poration	developing cortex	progenitors, early and late migrating neurons	IHC, mass spectrometry, microarray, ChIP	Chd3, Chd4, and Chd5 are mutually exclusive NuRD subunits during corticogenesis and regulate distinct set of genes; Chd4 promotes basal progenitor proliferation, Chd5 drives early radial migration, and Chd3 facilitates late migration and laminar specification.
(Muralidharan et al., 2017)	m	*Lhx2* knock-out, in utero electro-poration	developing cortex	deep layer 5 and 6, superficial layer 2 and 3	IHC, ISH, mass spectrometry, ChIP-seq/-PCR	*Lhx2*-null mice show more layer 5 neurons with high Fezf2/Ctip2 expression, while layer 6 neurons with Tbr-1 expression are less. Lxh2 regulates layer subtype specificity through enhanced recruitment of NuRD repressor activity to *Fezh2*, and its activator *Sox11*.
(Topark-Ngarm et al., 2006)	h	human neuroblastoma			transfections, chromatography, microarray	CTIP2 associates with NuRD on the promoter of *p57KIP2* and confers repression. shRNA-mediated knockdown of CTIP enhances *p57KIP2* expression.
(Harb et al., 2016)	m	*Lmo4* or *Nr2f1* knock-out, in utero electro-poration	postnatal cortex	somatosensory cortex, layer 5 projection neurons	IHC, ISH, ChIP, Co-IP, retrograde labeling	Ctip2/Satb2 co-expression defines two distinct subtypes of postnatal projection neurons. Thereby, Lmo4 targets Satb2/NuRD at *Ctip2* and prevents Hdac1-mediated histone deacetylation.

ChIP, chromatin immunoprecipitation; ChIP-seq, chromatin immunoprecipitation sequencing; Co-IP, coimmunoprecipitation; EM, electron microcopy; IHC, immunohistochemistry; ISH, in situ hybridization; qRT-PCR, quantitative reverse transcribed polymerase chain reaction; RNA-seq, RNA sequencing; shRNA, short hairpin RNA; WCPC, whole-cell patch clamp.

**Figure 2:**
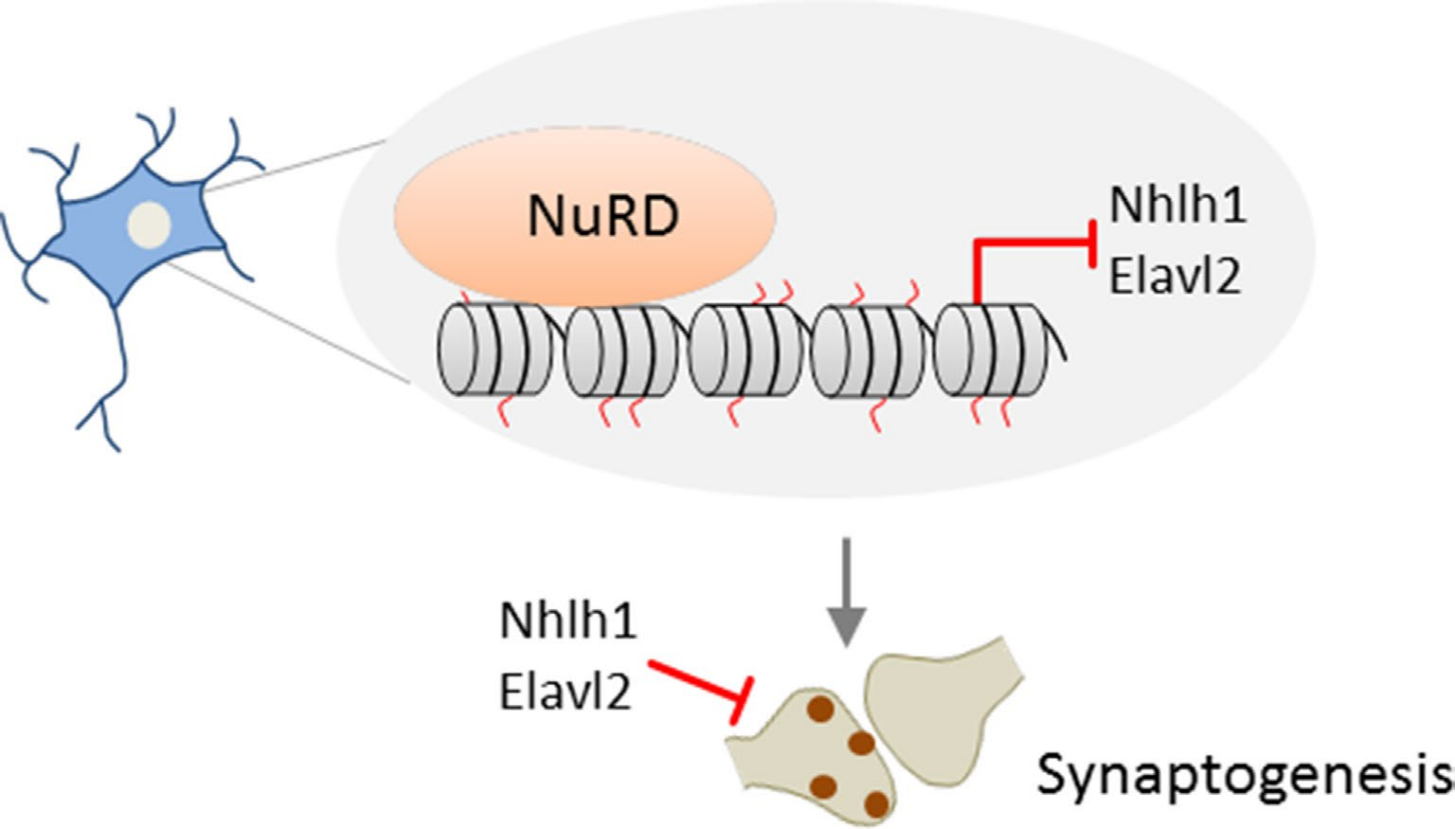
Promoter decommissioning by NuRD regulates presynaptic connectivity. In mice, repressive NuRD occupies a subset of genes during cerebellar cortex development. Among those genes, *Nhl1* and *Elav2* inhibit the development of granule neuron parallel fiber/Purkinje cell synapses. Model adapted from (Sun et al., 2014), license number 4578150712067.

Despite early evidence for NuRD’s role in development (see above), a role of NuRD in neuronal maturation has remained largely unexplored until recently. The formation of neuronal circuits depends critically on the differentiation of synapses in brain development and beyond (Kandel et al., 2013) and is shaped by both cell-intrinsic and environmental signals. In 2014, Yamada et al. (Yamada et al., 2014) first reported that NuRD regulates the differentiation of presynaptic sites in rodent cerebellum. *In vivo* RNAi and conditional *Chd4* knockout mice experiments showed that NuRD depletion in cerebellar cortex strongly impaired the development of granule neuron parallel fibers and of Purkinje cell synapses *in vivo*. Intersection of genome-wide RNA-seq and ChIP-seq data revealed a network of <200 repressed genes and decommissioned promoters at which NuRD turned off histone modifications associated with transcriptional activation during cerebellar development. A targeted *in vivo* RNAi screen of this network identified a subset of genes that encoded negative regulators of presynaptic differentiation: Nhlh1 (nescient helix loop helix) is a TF repressing the ubiquitously expressed bHLH factor TCF3 (*alias* E12/E47; E2A), and Elavl2 is an RNA-binding protein with a possible role in mRNA splicing and stability. Given the broad impact of these factors, they may serve as hub for NuRD’s effect on presynaptic development (**Figure 2**). In further support of and consistent with a role in presynaptic connectivity, NuRD operated throughout sensitive time windows of postnatal neuronal plasticity.

In a subsequent study, Yang et al. (2016) sought to explore through a multifaceted approach whether NuRD-dependent synapse development relates to distinct cerebellar functions. Genome-wide ChIP-seq showed that NuRD/Chd4-bound promoters belonged by and large to actively transcribed signaling genes in mice cerebellum [note that only a small network of <200 NuRD target genes previously associated with repressive histone modifications (Yamada et al., 2014)]. This result prompted Yang et al. to reason whether NuRD utilizes mechanisms distinct from posttranslational histone modifications to regulate gene expression. One such mechanism is the exchange of histone variants that are known to modulate transcription (Buschbeck and Hake, 2017). In support of this hypothesis, 97% of Chd4-bound active promoters were enriched in the variant H2A.z in wild-type, but not in conditional *Chd4* knockout mice. Combining RNA-seq with H2A.z ChIP-seq analyses revealed upregulation of >90% of the genes with reduced H2A.z occupancy, but little change in otherwise PTMs, in *Chd4*-null mice.

**Figure 3:**
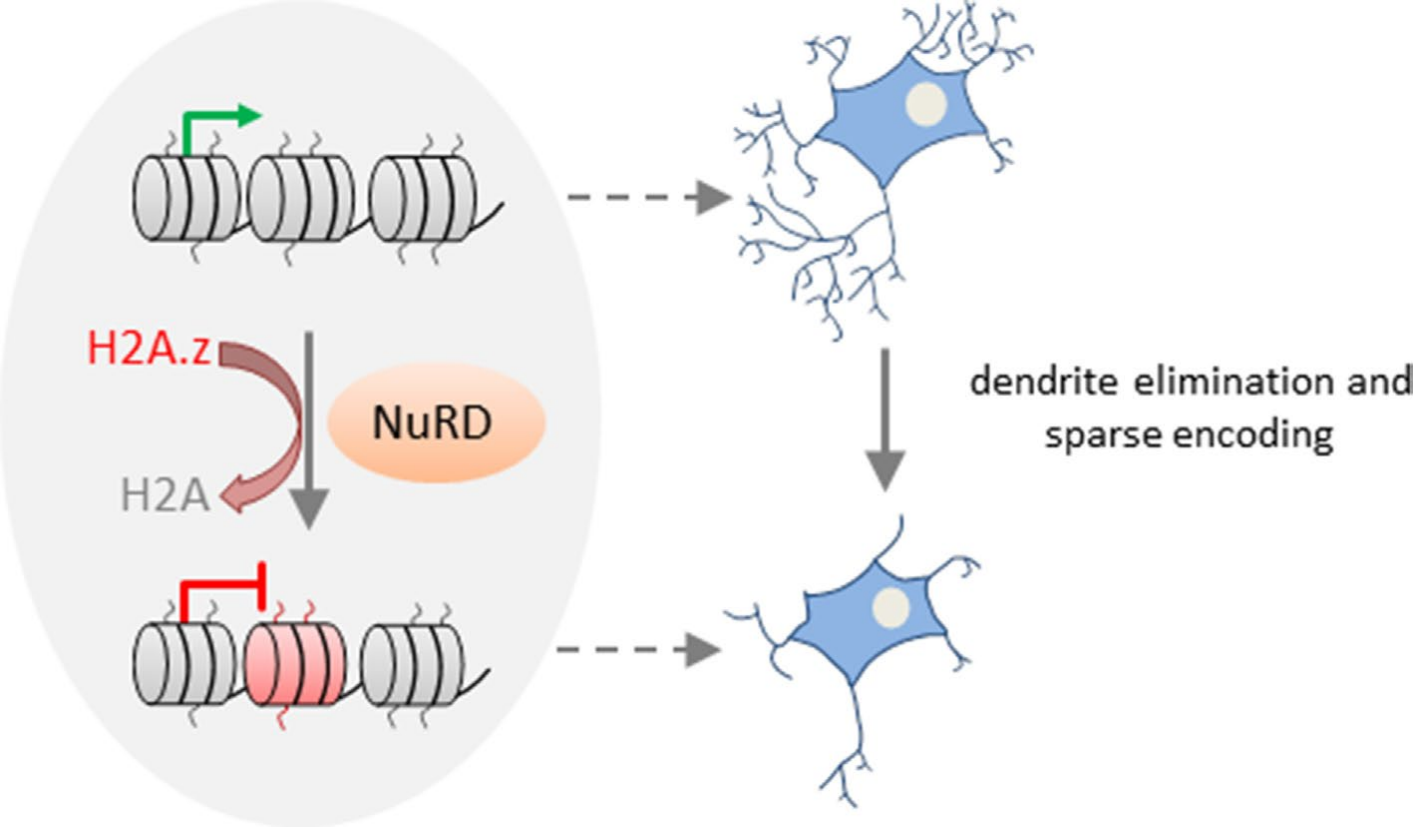
NuRD regulates activity-dependent transcription and neural circuit assembly and function. In cerebellar granule neurons, NuRD triggers deposition of the histone variant H2A.z (free histone ends shown in red) in exchange of the core histone H2A (free histone ends shown in gray) at neuronal-activity-dependent genes to reset transcription. NuRD-dependent modulation of gene expression changes contributes to synaptic pruning activity during sensitive developmental time periods. Thereby, NuRD fine-tunes cerebellar circuit function and promotes sparse encoding of information. Model adapted from (Yang et al., 2016), license number.4576500853137.

Together, these results suggest that NuRD mediates the replacement of the core histone H2A by the variant H2A.z *in vivo* at the promoters of a large group of neuronal-activity-dependent signaling genes in the cerebellar cortex and that deposition of H2A.z promotes gene deactivation (**Figure 3**).

Activity-dependent transcription is well known to modulate neuronal connectivity (Kandel et al., 2013). What is less known are mechanisms and functional implications of resetting activity-dependent transcription. Remarkably, neuronal activity enhanced deposition of H2A.z at NuRD-bound genes during the inactivation phase of transcription. Conversely, absence of NuRD slowed deactivation, although it retained the response to renewed neuronal activation. Thus, NuRD regulates dynamically neuronal activity-dependent transcription. By means of a rotating rod motor learning task, Yang et al. (2016) further showed that behavioral activity induced neuronal activity-dependent transcription *in vivo*, whose deactivation was slowed in NuRD-deficient mice. During the synchronous development of granule neurons *in vivo*, NuRD/H2A.z disabled activity-dependent gene expression and promoted dendritic patterning and connectivity. Conversely, the total length and number of primary dendrites declined in the absence of NuRD during the pruning period. This finding indicates that NuRD-mediated deactivation of neuronal-activity-dependent genes impacts pruning activity during sensitive time windows of granule neuron development (**Figure 3**). Such pruning processes are thought to fine-tune cerebellar circuit function and to enable sparse encoding of information. In support of this hypothesis, *Chd4*-null mice exposed to a sensorimotor stimulus (i.e., a treadmill task) showed hyperresponsive granule neuron activity *in vivo*. Such hyperresponsivity associated with impaired procedural learning capacity as evidenced in behavioral tests (i.e., accelerating rotarod and delayed eye-blink conditioning tests). By contrast, motor coordination was barely affected in *Chd4*-null mice.

Overall, these studies show NuRD-dependent chromatin remodeling in granule neuron synapse formation and connectivity during sensitive time windows. NuRD remodeling involves repression of a subgroup of inhibitory genes in synaptogenesis (**Figure 2**) and deactivation of a vast array of neuronal-activity-dependent signaling genes (**Figure 3**). While former process is more akin to NuRD’s silencer role, the latter process highlights NuRD’s role in constraining neuronal-activity-dependent gene expression changes that regulate granule neuron pruning activity and behavioral responses. In this respect, it would be interesting to know whether the deposition of the histone variant H2A.z at neuronal-activity-dependent genes is reversible and could respond to successive environmental exposures. While Yang et al. (2016) did not formally address this topic, it has been shown that the expression level and genomic deposition of histone variants are dynamically controlled (Buschbeck and Hake, 2017). Moreover, H2A.Z has been hypothesized to provide a means to dynamically increase chromatin accessibility and facilitate transitions between chromatin states. Along this line, H2A.Z has been assigned a role in nearly all functions of the chromatin template ranging from DNA repair and chromosome segregation to gene transcription and heterochromatin formation (Buschbeck and Hake, 2017). Taking these considerations into account, H2A.z deposition at neuronal-activity-dependent genes appears to be most likely reversible. Beyond PTMs, deposition and eviction of histone variants thus could add another layer of chromatin plasticity that impacts neuronal plasticity (Herre and Korb, 2019).

### NuRD in Cerebral Cortex Development and Maturation

**Figure 4:**
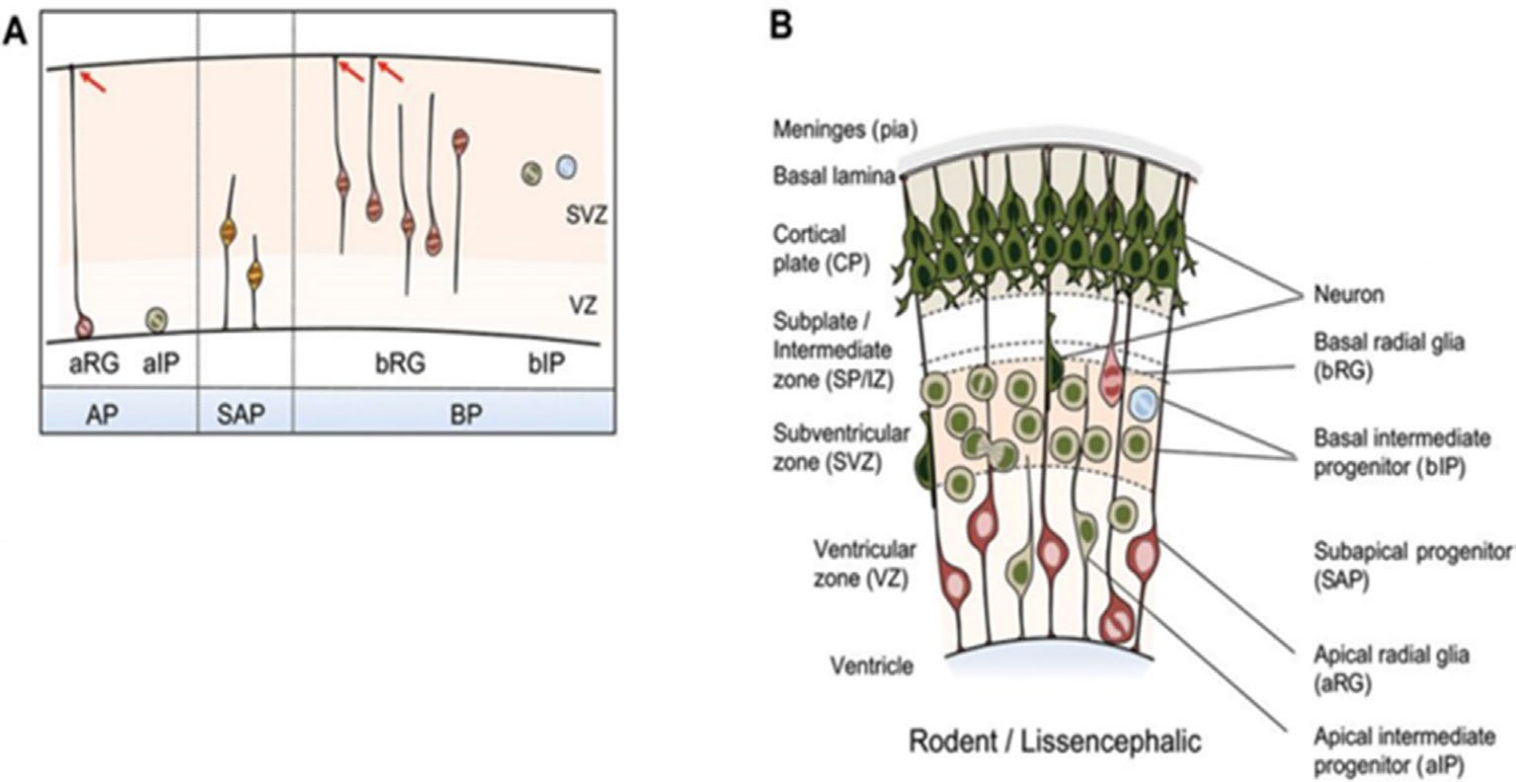
NPC types in the developing mammalian neocortex. **(A)** Neuroepithelial cells prevail prior to the onset of neurogenesis and are therefore not shown. NPCs are classified according to cell polarity, the presence of ventricular contact, and the location of mitosis. Apical progenitors (APs) comprise apical radial glia (aRG) and apical intermediate progenitors (aIPs). APs undergo mitosis at the ventricular surface in the presence of contact of the basal process with the basal lamina as indicated by red arrows. Subapical progenitors (SAPs) undergo mitosis at an abventricular location in the presence of ventricular contact. Basal progenitors (BPs) include basal radial glia (bRG) and basal intermediate progenitors (bIPs). BPs undergo mitosis at an abventricular location in the absence of ventricular contact. bRG subtypes are shown additionally: proliferative bIP *(blue circle)* and neurogenic bIP *(green circle)*. **(B)** Coronal section of the developing neocortex from mice. NPC types frequently found in each of the germinal zones are depicted. Schematic is partially from (Florio and Huttner, 2014), license number 4576520625938.

Formation of the highly organized cerebral cortex requires contributions from different classes of NPCs in a tightly spatiotemporally controlled sequence (Florio and Huttner, 2014). Key features of cortical progenitor cells and their role in cortical layer formation are schematically outlined in **Figures 4A, B**. Briefly, major groups of NPCs include apical progenitors (APs) and basal progenitors (BPs) that are classified according to cell polarity, presence of ventricular contact, location of mitosis, and the expression of astroglial markers. These NPCs undergo to different degrees self-renewing and differentiative cell divisions in the course of corticogenesis [interested readers are referred to Florio and Huttner (2014) for a comprehensive presentation].

Knock et al. (2015) first investigated a role of NuRD in the developing cerebral cortex in conditional *Mbd3* knockout mice. Because deletion of both *Mbd3* alleles is embryonic lethal, only heterozygous mice with unaffected viability were used. Macroscopically, heterozygous mice showed a reduction in cortical thickness that associated at the cellular level with an impaired specification of cortical projection neuron (PN) progenitors relative to wild-type mice: Pax6-positive APs were maintained in NuRD/Mbd3-mutant cortex but failed to respond to signals regulating symmetric versus asymmetric divisions and exited prematurely the cell cycle. Thereby, they produced insufficient amounts of Tbr2-positive BPs and neurons, and cortical plate neurons with deficits in terminal differentiation.

The mouse cortex develops between embryonic days 11 and 18 in a characteristic inside–out sequence: deep layers (designated 4–6) are formed first and upper, more superficial, layers (designated 2 and 3) are formed later. As a result, prospective upper layer neurons need to migrate through the deeper layer to attain their final destination (Florio and Huttner, 2014). Mbd3 was expressed in a subpopulation of cortical plate neurons that corresponded mainly to upper-layer neurons. In the absence of *Mbd3*, specification of upper layers (i.e., Satb2- and Brn2-positive neurons, see below) was compromised, and both deep and upper cortical layer markers became coexpressed. Expression profiling of microdissected tissues further revealed that NuRD/Mbd3 was necessary to guide neurodevelopmental differentiation programs by terminating the expression of proneural genes.

Briefly, this study indicates that NuRD/Mbd3 coordinates cerebral cell lineage choice and differentiation programs by terminating proneurogenic transcription in both progenitor and neural progeny.

**Figure 5:**
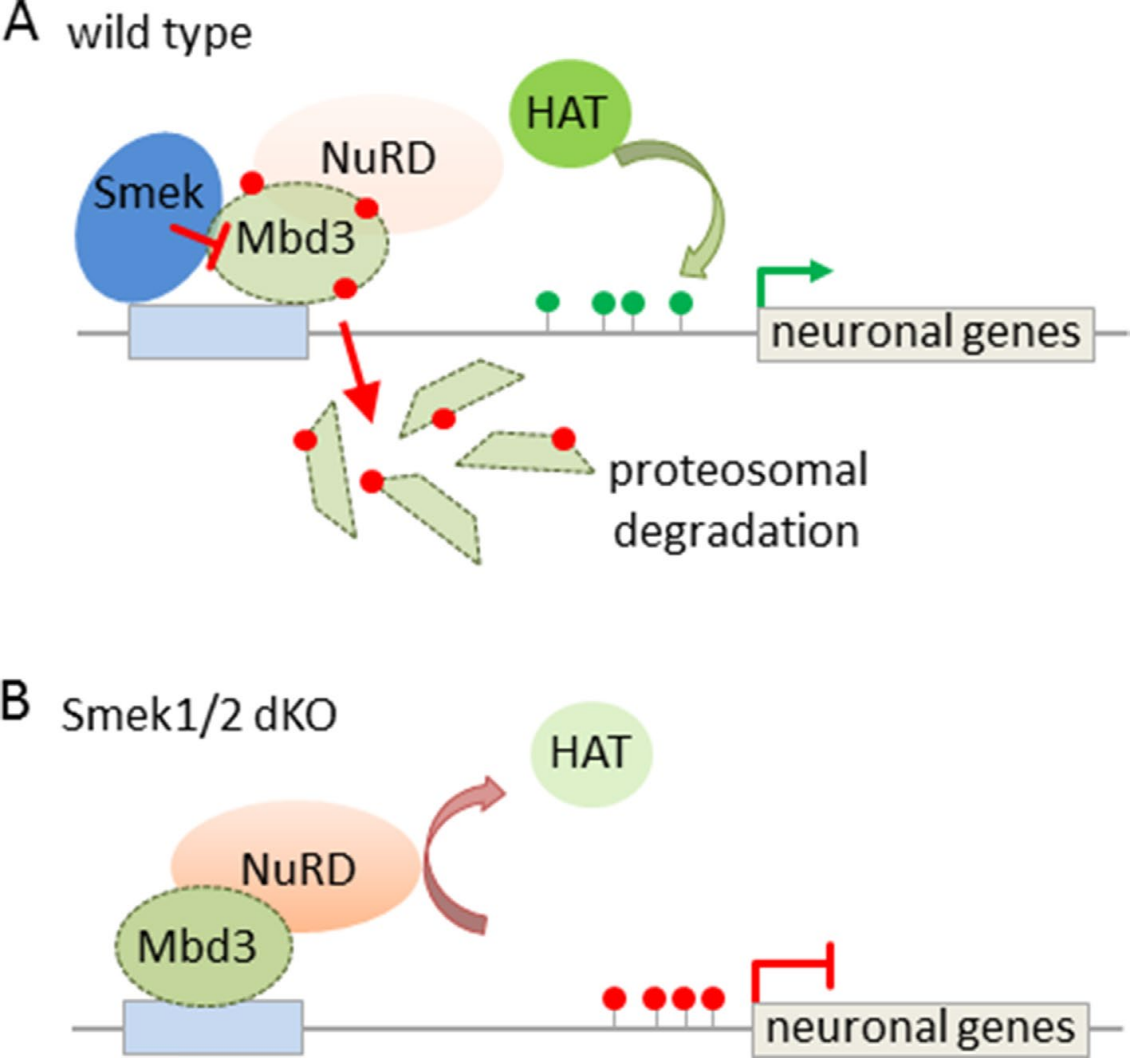
Interaction between Smek and NuRD/Mbd3 regulates NPC proliferation and fate. (A) Smek and Mbd3 co-localize at pro-neural/neural genes in cortical NPCs. Smek binding depends on the presence of Mbd3, and once bound, Smek promotes the polyubiquitylation (red dots) and degradation of Mbd3. This prevents NuRD formation and concurs with increased histone acetyltransferase (HAT) activity and active chromatin marks (green lollipops) driving neural/neuronal gene transcription. (B) Conversely, in Smek1/2 double knockout (dKO) mice, Mbd3 binding is maintained at pro-neural/neural genes in NPCs. This favors NuRD formation and confers gene repression by counteracting HAT activity. Model adapted from (Moon et al., 2017), attribution CC BY.

Subsequent studies have sought to identify mechanisms regulating NuRD’s activity in this process. In this regard, the suppressor of Mek-null (Smek) was found to bind to Mbd3 (Moon et al., 2017) and to inhibit the recruitment of NuRD at neurogenesis-associated gene loci (**Figure 5A**). Smek proteins are evolutionary conserved across eukaryotes and regulate asymmetric cell division in invertebrate neuroblasts. Two orthologous proteins, Smek1 and Smek2, exist in mice, and especially Smek1 enhances neuronal differentiation of NSCs by inhibiting Par3, an essential regulator for asymmetric cell division and polarized growth. Consistent with this finding, double knockout Smek1/2 mice showed defects in cortical neurogenesis *in vitro* and *in vivo* (**Figure 5B**). To gain insight in the underlying mechanism, Moon et al. (2017) conducted a yeast two hybrid screen that led to the isolation of Mbd3 as interaction partner of Smek. Further genome-wide profiling revealed Smek binding at genes relevant to brain development, differentiation, and cell-fate determination. Both Smek and Mbd3 colocalized at proneural/neural genes, where Smek binding depended on the presence of Mbd3. Once bound, Smek triggered polyubiquitylation and degradation of Mbd3 and thus reduced formation of and repression by NuRD/Mbd3 (**Figure 5A**).

Taken together, both Smek and Mbd3 tilt the balance between self-renewing and neurogenic cell divisions in the same direction. Since both proteins colocalize in NSCs, in which Smek counteracts NuRD/Mbd3, this findings raises the question to what degree Smek- and NuRD/Mbd3-dependent corticogenesis differ from each other.

In contrast to Mbd3, a role of Mbd2 in corticogenesis is still uncertain. Unlike most other MBD proteins, Mbd2 may be dispensable for brain function (Wood and Zhou, 2016). Alternatively, *Mbd2* may be required only in a small subpopulation of cells that do not manifest robust changes. In support of this hypothesis, proliferation and differentiation of olfactory receptor neurons were impaired in *Mbd2*-knockout mice (MacDonald et al., 2010), while olfaction-associated behavior was sustained. On the other hand, Lax et al. (2019) recently reported that *Mbd2*-knockout mice showed subtle deficits in cognitive, social, and emotional functions and downregulation of neuronal gene pathways in the adult hippocampus. Refined studies may help to define the contribution of NuRD/Mbd2 in neurodevelopment more precisely.

Apart from Mbd2/3, additional evidence suggests that distinct members of single NuRD subunits can serve as versatile regulatory mechanism during neurodevelopment. Of particular interest in this respect is NuRD’s ATPase activity, for which at least nine different genes have been identified so far. Most of these are expressed in neural cells through different stages of development (Micucci et al., 2015).

Unlike the cerebellum, Chd5, but not Chd4, was necessary for embryonic neocortical development in mice (Egan et al., 2013). While Chd5 was not expressed in rapidly proliferating progenitors, expression steadily increased in late-stage neuronal progenitors undergoing terminal differentiation. *In utero* knockdown of Chd5 resulted in a severe defect of progenitors to exit the germinal zones (ventricular, subventricular, and intermediate) and an accumulation of undifferentiated progenitors. In an ESC model of neurogenesis, Chd5-depleted cells failed to upregulate genes involved in late stage neural differentiation including synapse development, neuron projection, and neurotransmitter transport. At the same time, a subgroup of Polycomb target genes (Hoffmann et al., 2017b) underwent derepression in Chd5-depleted cells. Derepressed genes belonged to nonneuronal lineages including extraembryonic, mesodermal, and endodermal germ layers. Similar results were obtained in a cellular model of differentiated, Chd5-knockdown, neuroblastoma cells, which were analyzed by genome-wide ChIP-seq (Egan et al., 2013). Consistent with these findings, primary rat cortical neurons depleted of Chd5 showed alteration in the expression of neuron-specific genes and of other chromatin regulators such as the BAF complex (Potts et al., 2011).

Conclusively, terminal neural differentiation during embryonic corticogenesis requires Chd5’s two-sided function in gene regulation: to activate neuronal gene expression and to repress concurrently Polycomb-regulated genes controlling the expression of nonneuronal genes.

**Figure 6:**
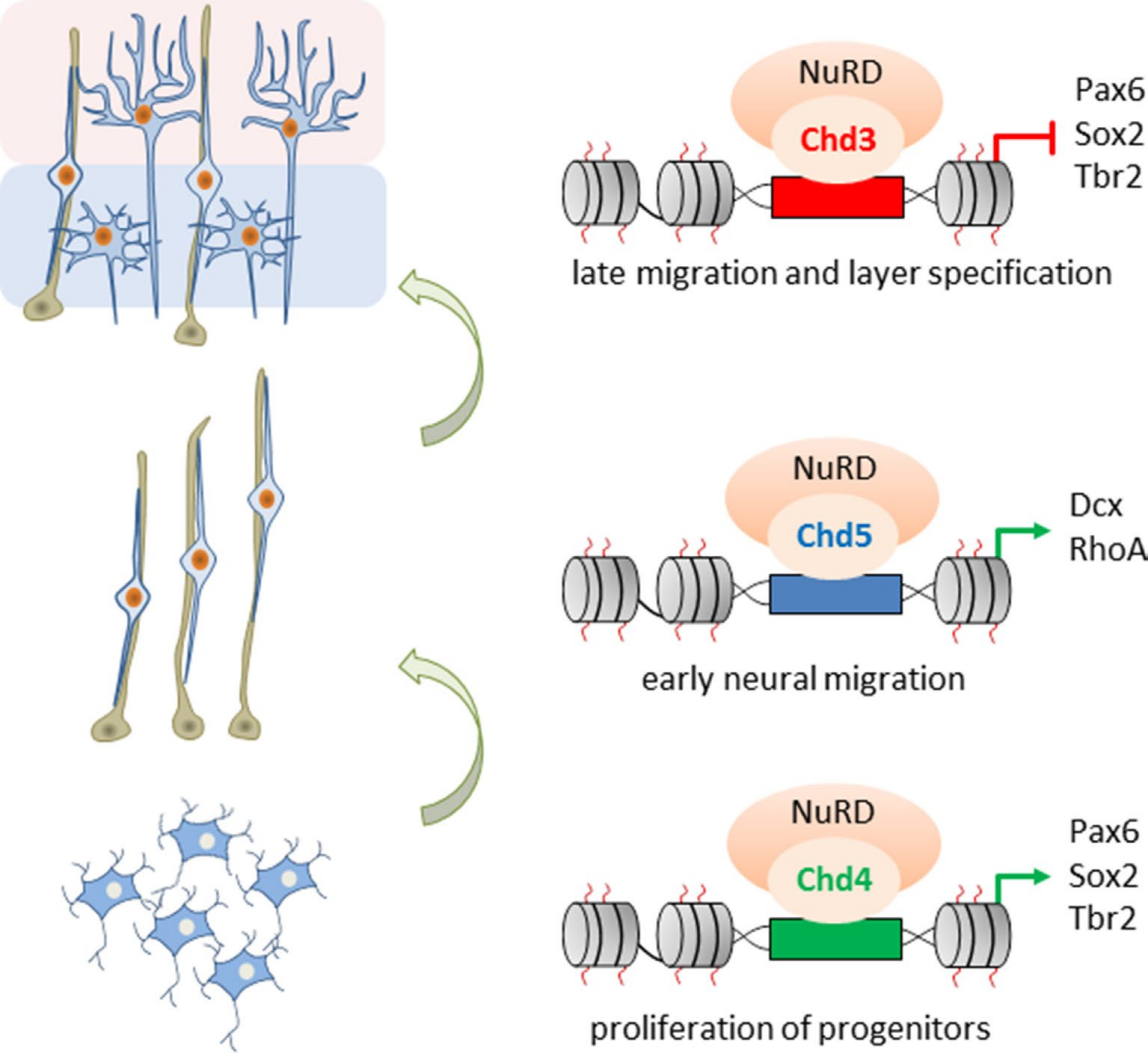
Role of Chd3/4/5 subunits in cerebral development. The ATPases Chd3/4/5 are mutually exclusive subunits of NuRD and regulate distinct and nonredundant aspects of mouse embryonic corticogenesis. Chd4 enhances proliferation of basal progenitors *(bottom)*, while Chd5 promotes early radial migration *(middle)*. In turn, Chd3 promotes late migration and specification of cortical neurons *(top)*. Model adapted from Nitarska et al. (2016), attribution CC BY.

Nitarska et al. (2016) went on to define NuRD’s role in distinct stages of neocortical development by mass spectrometry of Hdac2 imunoprecipitates. The ATPases Chd3/4/5 were present as mutually exclusive subunits of NuRD and regulated distinct set of genes essential for brain development: Chd4 promoted the proliferation of BPs, while both Chd5 and Chd3 promoted stage-specifically cell migration: Chd5 facilitated early radial migration, whereas Chd3 guided late migration of cortical neurons and regulated aditionally laminar specification (**Figure 6**).

NPCs depleted of *Chd3* exited the cell cycle prematurely and led to a subsequent deficit in BPs. This event reduced specifically the formation of upper layer neurons (Satb2- and Cux-1-positive) and finally of cortical thickness, a finding resembling Mbd3-deficient mice (Knock et al., 2015).

Contrary to Chd4, Chd3/5 expression was very low in NPCs (Nitarska et al., 2016) and increased continuously during late stages of neurogenesis. At this stage, neurons migrate radially to form the cortical plate. Neurons that had populated the cortical plate expressed Chd3, while neurons residing in the SVZ expressed additionally Chd5. *In utero* knockdown of Chd5 resulted in an accumulation of neurons in the intermediate zone and a failure to reach the cortical plate. In contrast, knockdown of Chd3 caused a delay in late neuronal migration: cells lagged behind in the deeper cortical layers and only a reduced number of neurons immigrated into the upper layers. Additional microarray and ChIP experiments indicated that such a sequential switch of Chd3/4/5 underpins distinct NuRD activities and confers transcriptional competence for the selective regulation of genes involved in the proliferation of neural progenitors, early radial and late cortical migration, and the specification of cortical layers.

Overall, these studies support a regulatory role of NuRD through consecutive building blocks of corticogenesis that requires distinct ATPase subunits and assign to NuRD a more dynamic function than originally thought. Mechanistically, different classes of neurodevelopmental TFs may need to interact with specific Chd subunits to fulfill their roles and thus present the driving force behind the exchange of Chd subunits in the course of neurodevelopment.

### NuRD Function in Neocortical Subtype Specification

High-level functions of the neocortex (e.g., cognition, articulation of language, sensory perception, and fine motor skills) are executed by excitatory PNs, presenting the largest portion of cortical neurons, and by inhibitory interneurons (Lodato and Arlotta, 2015).

**Figure 7:**
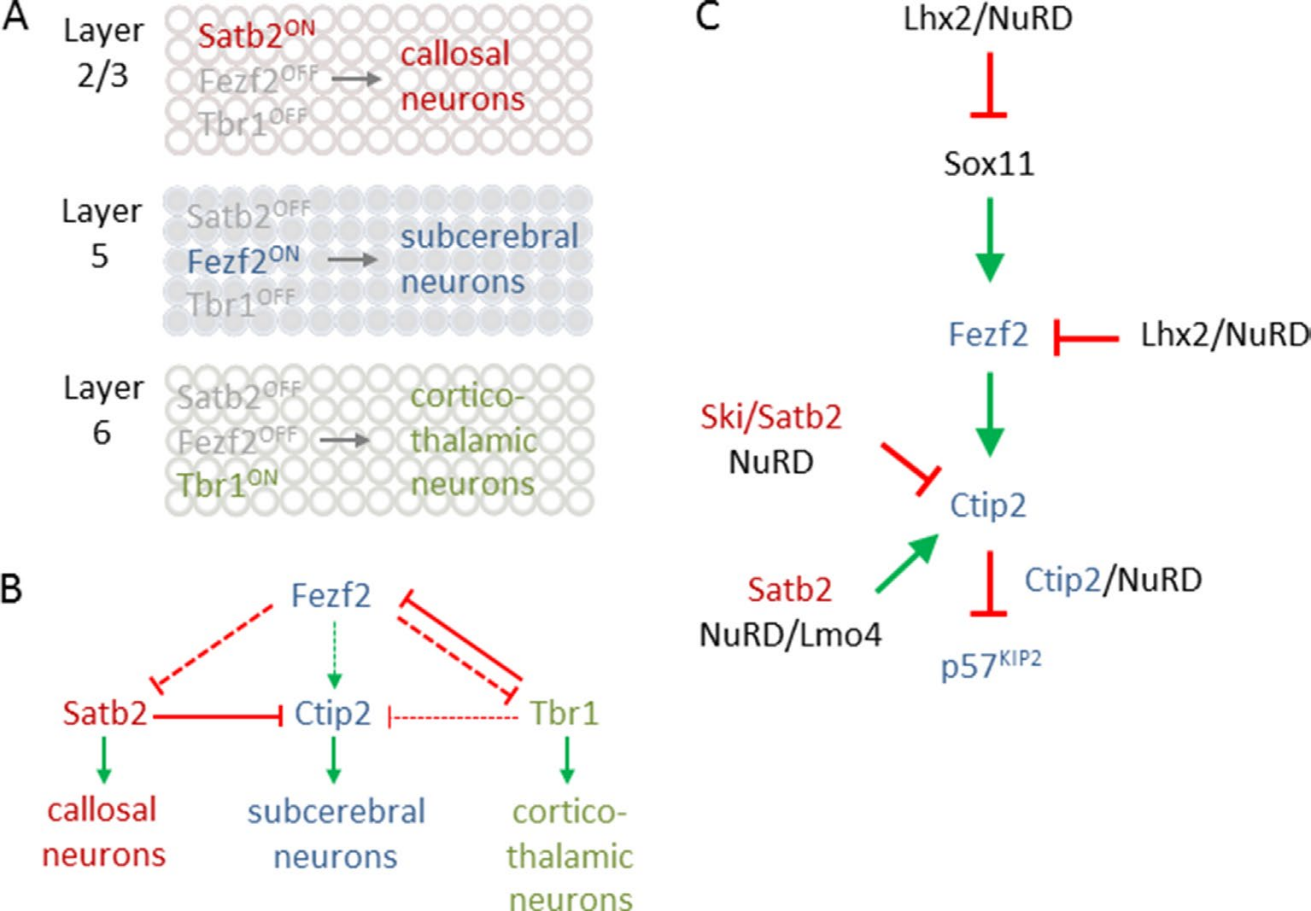
Transcriptional logic in cortical fate specification. **(A)** Scheme depicts key transcription factors for fate specification in different cortical layers. Satb2 expression in layer 2/3 defines callosal projection neuron (CPN) specification *(top)*. By contrast, Fezf2 expression in layer 5 determines subcerebral projection neuron (SCPN) specification *(middle)*, while Tbr1 expression in layer 6 is critical for directing fate divergence toward corticothalamic projection neuron (CThPN) specification *(bottom)*. **(B)** Scheme depicting the interactions between different key transcription factors for neuron identity specification. Arrows and bar-end lines indicate positive *(green)* or negative *(red)* indirect *(dashed)* or direct *(continuous)* regulation. **(C)** NuRD regulates expression of *Sox11*, *Fezf2*, and *Ctip2* as well as Ctip2 function, to fine-tune layer formation and postmitotic subtype specification. Green arrows and red bar-end lines indicate gene activation and repression, respectively. Schematic A and B is adapted from (Leyva-Díaz and López-Bendito, 2013), license number 4578091101882.

Excitatory PNs are classified into numerous subtypes based on three major criteria: first, by the location within six cortical layers that are defined by histological measures; second, by their axon projections to different intracortical, subcortical, and subcerebral regions; and third, by the expression of genes signifying specific subtypes of neurons (**Figure 7A**). In the context of NuRD’s role in cortical development, we focus here particularly on latter criteria.

The transcriptional logic underpinning specification of major classes of PNs including callosal PNs (CPNs), subcerebral PNs (SCPNs), and corticothalamic PNs (CThPNs) is schematically summarized in **Figure 7A** [interested readers are referred to Lodato and Arlotta (2015) for a comprehensive presentation]. In essence, PN identity evolves progressively by a fine-tuned transcriptional balance between genetic programs guiding the development of alternative types of PNs. Mechanistically, this balance involves cross-repression and cross-activation of key developmental regulators and extends also to their regulatory feedback loops (**Figure 7B**). The aggregated effects from this transcriptional logic are thought to sort postmitotic PNS into corticothalamic, subcerebral, or callosal fates. When discussing the role of NuRD in PN specification, we will adhere to the molecular logic of this transcriptional circuitry rather than the temporal order of the referred publications.

**Figure 8:**
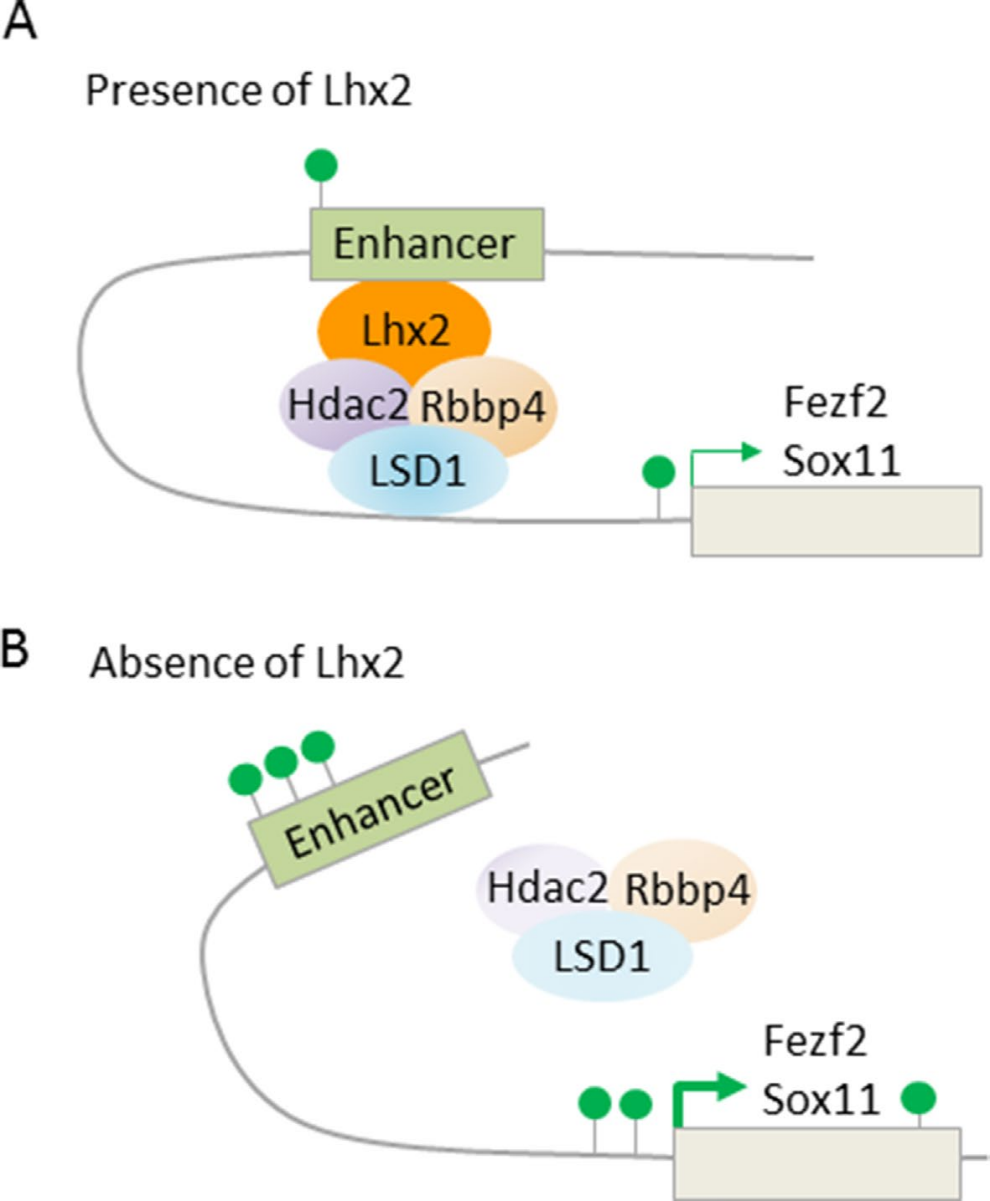
LHX2 binding at *Fezf2* and *Sox11* recruits NuRD subunits. **(A)** Scheme depicts Lhx2 binding to distal enhancers of the target genes *Fezf2* and *Sox11*. Lhx2 recruits repressive NuRD subunits and becomes juxtaposed to the transcription start site *via* chromatin looping. Consequently, transcription of *Fezf2* and *Sox11* is reduced with only few active histone marks *(green lollipops)* at the enhancer and transcription start site. Superficial layer neurons retain Lhx2 expression from their birth date through maturity, thus preventing subcerebral identity, while neurons of deep layer 5 and 6 rapidly repress Lhx2 expression, thus favoring subcerebral identity. **(B)** Cortex-specific Lhx2 knockout mice show derepression of *Fezf2* and *Sox11*with an increase in active histone marks. This leads to a strong increase in layer 5 neurons expressing high levels of Fezf2 and Ctip2, indicating subcerebral fate. Model adapted from Muralidharan et al. (2017), attribution CC BY.

Fezf2 is required for SCNP specification (**Figure 7A**), while Tbr1, and also SOX proteins (SRY box), including repressors Sox4 and Sox5, and activator Sox11, are regulators of Fezf2 expression (Kast and Levitt, 2019). Additionally, LHX2 (LIM homeobox TF 2) has been recently found to contribute to this regulatory pathway (Muralidharan et al., 2017). Superficial layer neurons retain Lhx2 expression from their birth date through maturity. By contrast, Lhx2 expression is rapidly downregulated in deep layer 5 and 6 neurons. Conversely, cortex-specific Lhx2 knockout mice showed a strong increase in layer 5 neurons expressing high levels of Fezf2 and Ctip2, indicating SCNP fate. Concurrently, Tbr1 expressing layer 6 neurons were reduced and led to cortex thinning in Lhx2 knockout mice. Further ChIP-seq experiments revealed that Lhx2 bound at distal regulatory elements present in Fezf2 and Sox11. Mass spectrometry of Lhx2 immunoprecipitates revealed the presence of NuRD subunits, including Rbbp4, Hdac2, and Lsd1, as Lhx2 binding partners. Together, these results suggest that Lhx2 regulates subtype specificity in deep layer 5 corticofugal PNs through enhanced recruitment of NuRD-repressor activity to the central regulator *Fezf2* and its upstream transactivator *Sox11* (**Figure 8**).

*Ctip2*, a downstream target to Fezf2, encodes a transcriptional repressor, which bind sequence specifically to DNA or interacts with other promoter-bound members of the COUP-TF family. Topark-Ngarm et al. (2006) showed that CTIP2 complexes from human neuroblastoma cells contained GATAD2A/B, MTA1/2, RBBP4/7, or HDAC1/2. These NuRD subunits were recruited in a CTIP2-dependent manner to a plasmid harboring the promoter region of *p57KIP2*, encoding a cyclin-dependent kinase inhibitor, and conferred repression (**Figure 7C**). Consistent with this finding, knockdown of CTIP2 in neuroblastoma cells enhanced expression of a number of genes, including p57KIP2.

In mice, deletion of *p57kip2* led to cortical hyperplasia during late embryogenesis and postnatal life (Mairet-Coello et al., 2012). Cell cycle re-entry of RGs and IPs was increased during early corticogenesis but decreased at middle stages. Consequently, deletion of *p57kip2* enhanced primarily layer 5–6 neuron production. Taken together, these findings indicate a role of Ctip2/NuRD in negative feedback control of deep layer neuron production *via p57kip2* repression.

**Figure 9:**
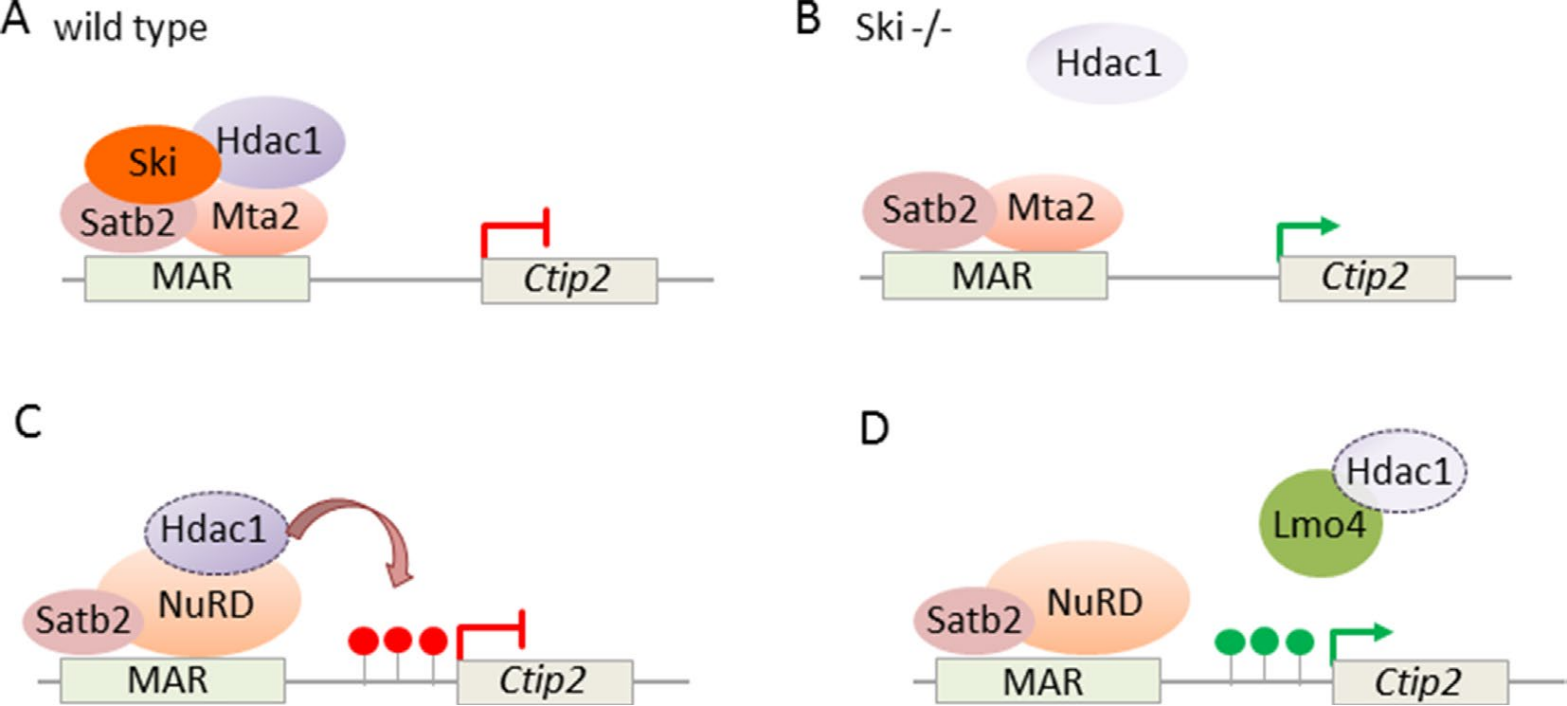
NuRD regulates *Ctip2* in upper layer cortical neurons. **(A)** Ski associates with Satb2 and represses *Ctip2* in callosal projection neurons. Ski is necessary to assemble functional NuRD repressor containing Satb2, Mta2, and Hdac1 at the regulatory MAR *(matrix attachment region)* in the *Ctip2* locus. **(B)** In the absence of Ski, Satb2, and Mta2 binding at the MAR is retained although Hdac1 recruitment is reduced. Model adapted from (Baranek et al., 2012), attribution CC BY. **(C)** NuRD regulates specification of projection neuron subtypes. During embryonic corticogenesis, NuRD binds at the regulatory MAR and represses *Ctip2* through Hdac1-mediated histone deacetylation *(red lollipops)*. **(D)** Lmo4 de-represses *Ctip2* in postmitotic projection neurons. Lmo4 regulates specification of Ctip2/Satb2-double positive neurons in layer 5 of the somatosensory cortex by interfering with Satb2-mediated *Ctip2* repression. Therefore, Lmo4 sequesters Hdac1 before it interacts with Satb2/NuRD in the *Ctip2* locus and thus maintains active histone marks *(green lollipops)*. Model adapted from Harb et al. (2016), attribution CC BY.

In addition, NuRD targets also directly *Ctip2*: expression of the transcriptional coregulator Ski (Ski sarcoma viral oncogene homologue) was high in postmitotic cells of the developing cortical plate in superficial layers (Baranek et al., 2012). Thereby, the expression of Ski closely resembled the one of Satb2. While *Ski* knockout mice showed relative to controls comparable thickness and cell numbers in cortical layers, Ski-deficient callosal neurons lost their identity and ectopically expressed Ctip2, albeit not Fezf2, indicating that they acquired some but not all characteristic of wild-type SCPNs. Since Ski-deficient callosal neurons phenocopied in part Satb2-deficient mice (Britanova et al., 2008), both factors seemed to operate in a shared genetic pathway. In support of this hypothesis, Ski and Satb2 interacted *in vitro* and *in situ* in upper layer neurons. Furthermore, *in vivo* ChIP experiments showed that Ski was recruited by Satb2 to previously identified Satb2-binding sites (known as matrix attachment region) in the *Ctip2* locus (**Figure 9A**). Consistent with previous findings (Britanova et al., 2008), Satb2 repressed *Ctip2* by recruiting NuRD *via* interacting with Hdac1 and Mta2. In the absence of Ski, the interaction of Satb2 with Mta2, but not with Hdac1, was retained, indicating Ski’s role to bridge Satb2 to Hdac1 (**Figure 9B**).

Briefly, Ski and Satb2 both target *Ctip2* in cortical CPNs and both proteins are required to maintain repression by NuRD in the *Ctip2* locus. Absence of either factor abrogates transcriptional repression and triggers partial loss of upper layer identity. As a whole, the picture emerging from these studies is that of a well-calibrated transcriptional logic of mutual “check and balances,” modulated by NuRD, in early specification of major classes of neocortical PNs.

On the other hand, our understanding of how PNs attain their final features during postnatal stages is still less advanced. In this regard, Harb et al. (2016) suggested recently an additional role of NuRD in the specification of PN subtypes. Although Ctip2 and Satb2 direct early specification of subcerebral and callosal PNs, respectively, their coexpression increases progressively in the postnatal somatosensory cortex. This coexpression of Ctip2 and Satb2 established two subtypes of layer 5 neurons, whereby one type projected to the contralateral cortex and the other type to the brainstem. These neuronal subtypes differed in their morphological and electrophysiological features from those neurons expressing solely Ctip2.

Ski expression was retained in Ctip2/Satb2 coexpression PNs, suggesting a mechanism independent of Ski to control the postnatal increase in Ctip2/Satb2 double positive somatosensory neurons. In support of this hypothesis, Lmo4 (Lim domain only), a transcriptional adaptor known to interact with several NuRD subunits, showed increasing peri- and postnatal expression in Ctip2/Satb2 coexpressing neurons. Overexpression and knockdown experiments demonstrated that Lmo4 acted in the specification of Ctip2/Satb2-double positive neurons primarily by modulating Ctip2 expression in layer 5: Lmo4 sequestered Hdac1 before its interaction with Satb2/NuRD and thus interfered progressively with Satb-2-dependent repression of *Ctip2* (**Figures 9C, D**).

In short, regulators such as Ctip2 and Satb2 with opposite function during embryonic corticogenesis can colocalize postnatally and contribute to the generation of diverse PN subtypes. Thereby, transcriptional adaptor Lmo4 targets Satb2/NuRD complexes in the *Ctip2* locus and promotes Ctip2 expression by interfering with NuRD-mediated deacetylation.

Overall, NuRD is a critical modulator of the molecular circuitry underlying specification of neocortical PNs in embryonic corticogenesis. Key regulators, such as Lhx2, Ctip2, and Satb2, increase the availability of NuRD at their target sites in a time- and area-specific manner to provide negative feedback control. Additionally, NuRD is a critical regulator of postnatal PN subtype specification through variation of molecular signatures that are shared with early corticogenesis. Similar connectivity or molecular code among neocortical areas has been hypothesized to originate, at least in part, from variations on a “common theme” rather than from the activity of many independent and region-specific genetic programs (Harris and Shepherd, 2015). Along this line, specific Chd subunits within NuRD have been previously shown to coordinate consecutive building blocks in neurogenesis. Similar variation in NuRD subunits, including and beyond Chds, may contribute as well to the specification of neocortical PNs. Future studies are needed to define NuRD subunit composition and the interaction with accessory subunits more precisely in this paradigm. In this respect, the aggregate effect from the interaction of tissue- or cell-type-specific TFs and NuRD subunits may present a critical determinant of molecular variation in postnatal specification of cortical neurons. This prompts also the question whether TFs and NuRD subunits may intersect in the regulation of their expression levels as to establish coherent transcriptional programs.

### A Role of NuRD in Neurodevelopmental Disorders

NDDs are complex conditions that result from anomalous brain development. Frequently, they present with impairments in multiple domains including cognition, language, social behavior and communication, and/or motor skills. ID, ASDs, attention deficit/hyperactivity disorder (ADHD), communication disorders, and SCZ fulfill the criteria of NDDs (American Psychiatric Association, 2013). Here, we consider genetic variation in NuRD subunits as risk factor for common polygenic forms of NDDs (with a focus on ID and ASD) and neurodevelopment-related psychiatric disorders (with a focus on SCZ and BD).

#### Role of NuRD Subunits in NDDs

A newborn carries on an average between 50 and 100 genetic variants. This number corresponds to 0.86 new amino acid altering mutations (i.e., *de novo* mutations) per individual (Lynch, 2010). Errors in DNA replication, which escape proofreading mechanisms, or errors in recombination are the major source of *de novo* mutations. They can arise already during parental gamete formation or at early stages of embryonic development. These variants can range from single nucleotide polymorphisms (SNPs) to gain or loss of large DNA regions comprising thousands of nucleotides [i.e., insertions or deletions and copy number variants (CNV)].

Prevalence rates for ID reach 1–3% (Srivastava and Schwartz, 2014) and comprise a group of disorders that present broadly varying clinical phenotypes. *De novo* loss-of-function mutations in *GATAD2B* were first identified by de Ligt et al. (2012) in 2 patients out of 100 with severe ID (IQ < 50) using whole-exome sequencing. Additional database searches and resequencing of *GATAD2B* identified another individual with a loss-of-function mutation and one individual with a microdeletion (Willemsen et al., 2013). Following these index cases, subsequent exome sequencing studies have detected additional *de novo* splicing and loss of function mutations (Hamdan et al., 2014; Vanderver et al., 2016; Luo et al., 2017; Ueda et al., 2018). Common clinical symptoms among these individuals include disorders of the eye (hyperopia. strabismus, and a hypoplastic optic nerve) and behavioral symptoms (hyperactivity, tics, and inappropriate laughter, wandering at night, and poor frustration tolerance concurrent with reduced social and communicative function).

There is at present only sparse information from animal models on the role of Gatad2b in neurodevelopment. In *Drosophila*, targeted knockdown of the Gatad2b ortholog in neurons showed impaired habituation, suggesting a defect in classic learning and memory (Willemsen et al., 2013). While these findings are consistent with a role of GATAD2B in human ID, further studies are needed to define cellular and molecular roles of this factor in neurodevelopment and brain function more precisely. Along this line, it remains to be clarified to what degree GATAD2A and GATAD2B are interchangeable in NuRD (see also below).

In contrast to GATAD2A/B, increasing evidence has accumulated for specific roles of CHD3/4 in neural progenitor proliferation, late neuronal migration, and cortical layer specification (**Figure 6**).

In a candidate approach, Weiss et al. (2016) detected in five individuals with developmental delay *de novo* missense substitution in *CHD4* using exome sequencing. These individuals shared additionally mild to moderate ID, macrocephaly, hearing loss, distinct facial dysmorphisms, palatal abnormalities, ventriculomegaly, and hypogonadism. All of the identified missense mutations localized to evolutionary highly conserved amino acid residues, which have been predicted to disrupt function. Three mutations localized in the C-terminal ATPase domain known to interact with HDAC1/2; however, this interaction was undisturbed as assessed by coimmunoprecipitation experiments. Hence, these missense variations were more likely to affect ATPase catalytic activity rather than NuRD formation. In support of this hypothesis, tumor-associated missense mutations in CHD4’s ATPase domain were recently found to be compromised for nucleosome remodeling activity (Kovač et al., 2018). Moreover, missense mutations close to the ATPase domain showed the opposite effect, indicating that CHD4 mutations could both decrease and increase NuRD-mediated nucleosome remodeling activity in NDDs.

Similarly to CHD4, the ATPase/helicase domain of CHD3 has been implicated in NDDs based on a candidate approach. Snijders Blok et al. (2018) detected through whole genome sequencing a *de novo* missense mutation in *CHD3* in a cohort of unrelated children (*N* = 19) with a primary diagnosis of childhood apraxia of speech. This mutation has been predicted to disrupt CHD3’s helicase domain. Given this index case, the researches went on to apply a genotype-based strategy to identify additional unrelated individuals (*N* = 35) with *de novo* mutations in *CHD3*. All of them shared global developmental delay and/or ID. At 2 years age or older, most of them showed a delayed development of speech and language. About half of the carriers presented macrocephaly, about one-third autism or autism-like features, and further symptoms including widening of cerebrospinal fluid space, hypotonic, and distinct facial dysmorphisms. The majority of the mutations clustered within highly conserved residues of the ATPase/helicase domain and was predicted to disrupt motifs critical to substrate binding and interaction. Among six of the identified mutations, a subset showed impaired ATPase activity *in vitro*, and five were impaired in chromatin remodeling. Briefly, *de novo* mutations in *CHD3* cause a syndrome featuring ID, impairments in speech and language, and macrocephaly. Well-fitting, cognitive capabilities depend on cortical functions that are under the control of NuRD/CHD3 during corticogenesis.

Owing to the steady progress in next-generation sequencing (i.e., whole-genome and whole-exome sequencing), more than 800 ID-related genes have been identified by now (O’Roak et al., 2012; Sanders et al., 2012; De Rubeis et al., 2014; Iossifov et al., 2014; Krumm et al., 2015; Yuen et al., 2015; Deciphering Developmental Disorders Study, 2017; Geisheker et al., 2017; Eising et al., 2018). These studies have detected many inherited and *de novo* germline mutations that significant impact total NDD risk and thus present novel disease genes. Moreover, the same mutations were shared in a sizeable fraction of patients presenting the same or similar disorders. This suggests that these mutations increase in general risk for abnormal brain development, whereby the eventual phenotype reflects the interaction with each carrier’s genetic background and/or environmental exposures (The Deciphering Developmental Disorders Study et al., 2014; Deciphering Developmental Disorders Study, 2017; Geisheker et al., 2017).

Many of the genes identified by these studies encode proteins for the regulation of transcription and chromatin remodeling, including *de novo* mutations of the NuRD core subunits *CHD3/4* and *GATAD2B*, and for synapse formation and plasticity (Deciphering Developmental Disorders Study, 2017; Eising et al., 2018). *De novo* mutations in NuRD subunits clearly exceeded the threshold (*P* < 7 × 10^e−7^) for genome-wide significant association for NDD. Of further note, *de novo* mutations were also identified in NuRD-regulated genes driving cortical layer formation such as *TBR1* and *SATB2* (Iossifov et al., 2014; Deciphering Developmental Disorders Study, 2017; Geisheker et al., 2017) with *SATB2* exceeding the threshold for genome-wide significance.

Most recently, Coe et al. (2019) integrated in a meta-analysis *de novo* exome mutations from cases with ASD, ID, and/or developmental delay with CNV morbidity data. This comprehensive analysis identified a group of candidate neurodevelopmental disease genes (*N* = 253) that were enriched for missense and/or likely gene-disruptive mutation. About half of these genes (*N* = 124) reached exome-wide significance (*P* < 5 × 10^e−7^) including the NuRD core subunits *CHD3/4* and the NuRD-regulated genes *TBR1* and *SATB2*, arguing that these genes contribute significantly to disease risk.

By now, few studies have sought to analyze molecular pathways in addition to sequence in order to gain insight into signaling processes, cell types, and neural circuits that underpin alterations in cognition and behavior in NDDs. For example, exome sequencing in a cohort of parent–child trios with sporadic ASD detected 126 severe or disruptive *de novo* mutations including *CHD3*, *CHD5*, and *TBR1* (O’Roak et al., 2012). About 39% of these genes, including *CHD3*, formed a β-catenin/chromatin-remodeling-protein network based on a database of physical interactions. Notably, β-catenin regulates Wingless signaling that plays a critical role in NDD risk including ID and ASD (Kwan et al., 2016). Likewise, network enrichment analysis identified four modules (Coe et al., 2019), among which module 3, including GATAD2B, highlighted the “transmembrane receptor protein serine/threonine kinase signaling pathway” that plays an important role in neurodevelopment and differentiation.

In an orthogonal approach to modeling genetic variation, Li et al. (2015) investigated the role of ASD candidate genes through a system-level approach. Specifically, they mapped the encoded proteins onto ubiquitous protein complexes isolated from human cell lines. Mass spectrometry analysis of immunoprecipitates revealed that ASD proteins were particularly enriched in complexes formed with macromolecular BAF and NuRD. Furthermore, Li et al. conducted proteome-wide screens in human neuronal cells for subunits co-complexed with HDAC1 and six crucial ASD proteins and thus identified a protein interaction network that was preferentially expressed in fetal brain development. This fetal network was enriched in deleterious mutations from ASD and genes underpinning monogenic forms of NDDs (e.g., fragile X and Rett syndrome). Collectively, this approach supports a role of BAF and NuRD in ASD and fetal brain development and further suggests shared mechanisms between syndromic and idiopathic forms of ASD.

Taken together, *de novo* mutations in NuRD core subunits and in NuRD-regulated genes present crucial risk factors in polygenic NDDs, which are shared among different clinical phenotypes. This indicates that genetic variation in NuRD-dependent chromatin remodeling in early brain development may lead to a vulnerable brain from which different kind of NDDs emerge in a manner dependent on neurodevelopmental time windows, genetic background, and the interaction with the environment.

#### Role of NuRD Subunits in Neurodevelopment-Related Psychiatric Disorders

Major psychotic disorders comprise SCZ, BD, and major depression (MD). Both SCZ and BD are hypothesized to arise, at least in part, from abnormal neurodevelopment. Here, we discuss recent evidence for a potential role of NuRD in these perturbations.

##### NuRD in SCZ

SCZ is a highly heritable devastating mental disorder (Sullivan et al., 2012) with a lifetime prevalence of ≈1% worldwide (World Health Organization, 2016). Clinical hallmarks comprise distortions in perception, thinking, and language together with impairment in emotion, sense of self, and behavior. Subtle perturbations in early neurodevelopment mediated through incompletely understood genetic risk factors are thought to increase later susceptibility for SCZ that unfolds in adolescence to early adulthood.

A landmark genome-wide association study (GWAS) meta-analysis (Ripke et al., 2014) has identified 128 genome-wide (*P* < 5 × 10^e−8^) significant associations comprising 108 independent loci, of which 93 have been recently replicated (Pardiñas et al., 2018). These 108 loci contain some 350 genes enriched in genes relevant to glutamatergic neurotransmission, neuronal ion channels, neuronal calcium signaling, synapse formation and plasticity, G-protein coupled receptor signaling, and neurodevelopmental regulators.

Whitton et al. (2016) reasoned whether any genes at current SCZ risk loci belong to chromatin regulators of gene expression and whether they associate independently with cognitive function. The researchers compiled a list of 350 unique chromatin-modulating genes, which were cross-referenced with the genes located in the 108 chromosomal risk regions in SCZ. This approach identified a shortlist of 17 “epigenetic regulators”: for seven genes, localized the associated single SNPs within or close to the gene location (*CTIP2*, *EP300*, *EPC2*, *GATAD2A*, *KDM3B*, *RERE*, and *SATB2*). Furthermore, three of these genes (*CTIP2*, *EPC2*, and *SATB2*) presented the only gene in the index region. Four variants (in *EP300*, *GATAD2A*, *KDM3B*, and *RERE*) showed nominally significant association with one or several cognitive task, while the risk allele for *GATAD2A* associated also with lower “Full Scale IQ.”

Collectively, this study indicates that genetic variation in the NuRD core subunit GATAD2A or in the NuRD-interacting regulators CTIP2 and SATB2 contributes to cognitive impairment in SCZ.

In a related approach, Ma et al. (2018) sought to systematically predict plausible candidate genes for SCZ in the 108 risk loci through a comprehensive integrative analysis of different prediction approaches with a focus on brain-specific shared-function or cofunction networks. This analysis detected a group of candidates comprising *CNTN4*, *GATAD2A*, *GPM6A*, *MMP16*, *PSMA4*, and *TCF4*. Consistent with this finding, four of these top candidates, including *GATAD2A*, have been also previously identified as SCZ risk gene (Hauberg et al., 2017) through integrative analysis of GWAS and eQTL data in various tissues. Additional cell-type-specific expression analysis indicated that these top candidates and a set of additional high confidence candidates (including *CTIP2*) were significantly higher expressed in neurons than in oligodendrocytes and microglia. This gene set also formed a densely interconnected protein–protein interaction network enriched in synaptic neurotransmission-related pathways. In postmortem hippocampus, the expression of GATAD2A and TCF4 was enhanced in cases with SCZ relative to controls. Functionally, shRNA-mediated knockdown of the six top candidate genes *in vitro* impaired proliferation of neuroblastoma cells.

Taken together, GWAS and integrative pathway/biostatistics analyses indicate a role for genetic variation in NuRD subunit *GATAD2A* and in NuRD-regulated *SATB2* and *CTIP2* in SCZ/cognition.

##### NuRD in BD

BD presents extreme mood swings with mood-congruent delusions (Strakowski, 2012). Sleep disturbances often precede relapse and contribute to mood disruption. Similarly to SCZ, BD is highly heritable and manifests in adolescence or early adulthood. Current meta-analyses of GWAS showed that >40 genes associated with susceptibility to BD (Ikeda et al., 2017). Furthermore, recent GWAS also support a genetic correlation near 0.6–0.7 between BD and SCZ as inferred from common genetic variation (Cross-Disorder Group of the Psychiatric Genomics Consortium et al., 2013).

Genetic variation in circadian genes, also known as clock genes, has been associated with different mental disorders, especially BD (Oliveira et al., 2018) and autism (Nicholas et al., 2007). The molecular clock consists of a conserved negative transcriptional feedback loop, which builds on three PER and two CRYPTOCHROME (CRY) proteins. Following their accumulation, they form a large nuclear complex that serves as a scaffold for the recruitment of different effector proteins that repress transcription. This complex binds directly to DNA-bound TF CLOCK-BMAL1 and confers repression onto specific target genes, including PER and CRY. In consequence, clock genes drive the expression of hundreds of genes in central and peripheral cells in a rhythmic fashion.

**Figure 10:**
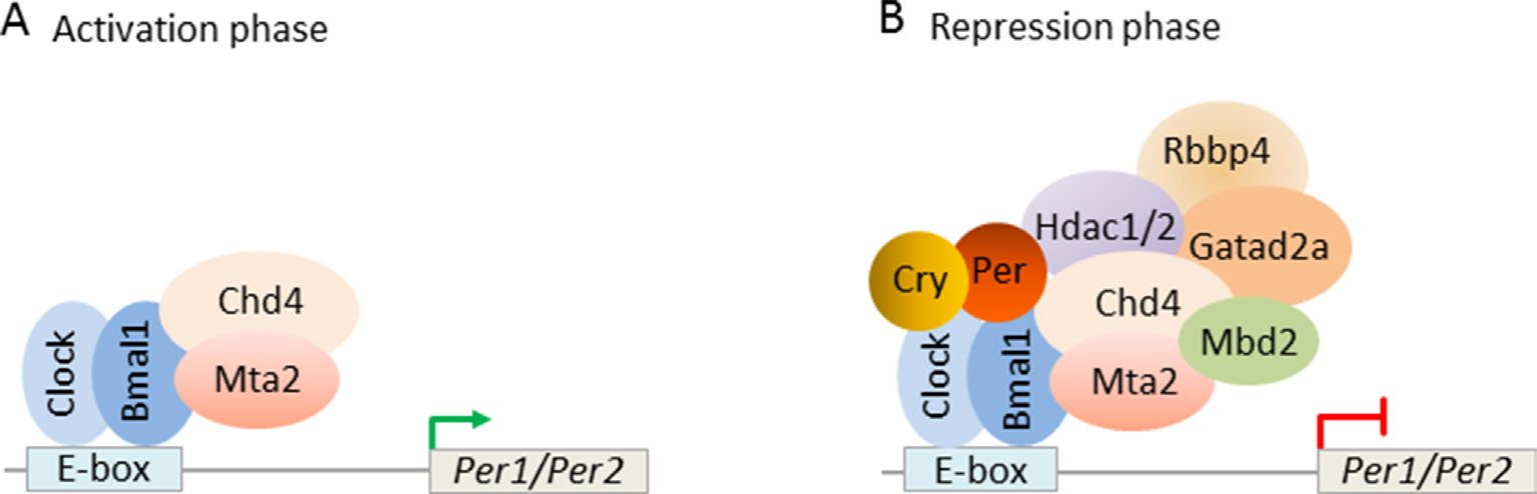
Circadian clock feedback involves targeted reconstitution of NuRD by the PER complex. **(A)** During the circadian transcriptional activation phase, the transcription factors Clock-Bmal1 assemble with the NuRD subunits Chd4 and Mta2 at the E-box of the circadian target genes *Per1/Per2*. Under this condition, Chd4 promotes Clock-Bmal1 transcriptional activity. Mta2 is necessary for the subsequent assembly of NuRD repressor. **(B)** During the circadian negative feedback phase, newly formed PER complex brings the remaining NuRD components to Clock-Bmal1 at the E-box and reconstitutes functional NuRD repressor. Repressor activity of the PER complex thus depends on correct targeting of Clock-Bmal1. Model adapted from Kim et al. (2014), license number 4578121170114.

Interestingly, Kim et al. (2014) found that mouse PER complexes stepwise associate with NuRD to induce a transcriptional switch: first, Clock-Bmal1 bound constitutively to Chd4 and Mta2 and enhanced the transcriptional activity of Clock-Bmal1 (**Figure 10A**). Second, with the beginning of negative feedback, the PER complex recruited the residual NuRD subunits to DNA-bound Clock-Bmal1 and thus reconstituted NuRD-mediated repression of clock genes. These data reveal that repressive NuRD is initially partitioned between Clock-Bmal1 and the emergent PER complex and that pending PER’s association with DNA-bound Clock-Bmal1 functional NuRD is site-specifically reconstituted (**Figure 10B**).

Apart from its role in the molecular clock, NuRD has been also implicated in early brain development in BD by systematic analysis of GWAS data. Xiang et al. (2018) integrated GWAS for BD to establish first a gene network of significant pathways. In a second step, they intersected this network with a gene set analysis of each gene cluster identified by ENIGMA (a neuroimaging GWAS study). This approach identified 30 pathways and 22 interconnected functional and topological interacting clusters that associated with BD risk. Further intersection with brain transcriptome datasets (BrainSpan) showed significant associations with common variants in cluster 1 for the hippocampus and amygdala. Cluster 1 comprised the core genes *CHD4*, *MTA2*, *RBBP4*, and *HDAC2*, all of which encode NuRD subunits. This cluster was also enriched for coexpressed genes regulating prenatal amygdala development. Collectively, this work indicates a critical function of the hippocampus and amygdala in brain development and associated BD risk and implicates NuRD in these processes.

A recent study (Bipolar Disorder and Schizophrenia Working Group of the Psychiatric Genomics Consortium, 2018) utilized a large collection of genotyped samples for BD (*N* = 20,129) and SCZ (*N* = 333,426) together with clinically relevant data to identify 114 genome-wide significant loci that were shared between these disorders and were enriched in genes underpinning synaptic and neuronal pathways. An additional comparison between SCZ and BD identified four genomic regions that contributed to differences in their biology. Combined regional association and heritability estimates were used to assess the contribution of these genomic regions to each disorder. This analysis suggested a locus that contributed differentially to vulnerability to BD and SCZ: although the association peaks of both disorders overlapped at this locus, they originated from independent causal variants for each condition. Interestingly, the same gene, *GATAD2A*, was significantly regulated by these variants though in opposite directions. As noted before (Li et al., 2015), *GATAD2A* is preferentially expressed in fetal brain development. Hence, genetic variation tilting the balance of NuRD activity in either direction in neurodevelopment, particularly in corticogenesis, may increase risk in SCZ or BD.

Taken together, mutations in NuRD subunits are highly significantly associated with NDDs (e.g., CHD3*/4* and *GATAD2B*) and neurodevelopment-related psychiatric disorders (e.g., *GATAD2A*). Owing to the nature of polygenic diseases, in which numerous variants are thought to contribute incrementally to risk, mutations in NuRD subunits will have only minute effects at the level of single variants. However, sufficiently empowered patient-specific iPSC studies (Ahmad et al., 2018) will offer the opportunity to dissect the regulatory effects of these variants on the development and function of living human neurons in order to advance our insight into the molecular and cellular foundations of these conditions.

## Discussion and Outlook

NuRD presents an important epigenetic regulator of gene expression in NSC and NPC fate decisions in cortical brain development. Postmitotically, NuRD controls additionally synaptic plasticity, neuronal connectivity, and neuronal subtype specification. Consistent with these activities, genetic variations in these genes are important risk factors in common polygenic forms of NDDs and neurodevelopment-related psychiatric disorders such as SCZ and BD. Overall, these findings highlight the role of NuRD in chromatin regulation in brain development, and in mental health and disease.

Biochemical and genetic studies have shown that NuRD combines ATPase/helicase and histone deacetylation activities in chromatin remodeling. A diversity of NuRD subunits together with a vast array of tissue-specific factors and multifarious TFs contribute to this dual activity and the cooperation with other chromatin modifying complexes. Notably, almost all of these studies have built on tractable cellular models, particularly ESCs, as to tackle the complexity of NuRD subunits, their interactions and functions, and the mode of chromatin binding. This ground laying work has advanced substantially our view of NuRD and has drawn a more dynamic picture of NuRD integrating both repressive and activating features. Along these lines, NuRD may control gene expression changes by modulating chromatin plasticity rather than by imposing categorical “on–off switches” in gene regulation. As a case in point, NuRD plays a critical role in resetting neuronal-activity-dependent gene expression changes in developing cerebellar neurons with important implications for sparse information processing (Yang et al., 2016).

As much as we know about NuRD in well-defined cellular models, the composition and function of this macromolecular complex *in vivo*, particularly in human brain, are much less understood. Recent evidence in mice suggests that the ATPase/helicase subcomplex may operate independently of NuRD in early embryonic development (O’Shaughnessy-Kirwan et al., 2015). Moreover, targeted reconstitution of NuRD has been described in circadian transcriptional feedback (Kim et al., 2014). Thus, NuRD subcomplexes may also operate either in isolation or come together as part of a regulatory process controlling *in vivo* NuRD activity. These findings highlight the complexity of NuRD and make further *in vivo* studies necessary to deepen our insight into its assembly and into signals governing its association with specific cofactors as well as its activity. In this context, we would like also to caution that several NuRD subunits fulfill distinct roles outside of NuRD (e.g., MBDs) and/or assemble in other macromolecular complexes (e.g., HDACs). Hence, NuRD’s *bona fide* involvement in gene regulation needs to be carefully assessed both in terms of the presence of critical core subunits and their actual assembly *in vivo*. Relatedly, single component knockouts and mutations might not only impact NuRD, for example by destabilizing other subunits, but may also have secondary effects due to a genuine role of the respective subunit outside of NuRD.

Emerging evidence indicates that tissue- and cell-type-specific TFs interact with specific NuRD subunits in a spatiotemporal defined manner during neurodevelopment and beyond. Such diversity in the composition of NuRD subunits could serve to constrain NuRD function to distinct neurodevelopmental time windows as to fulfill distinct roles in cell lineage specification, neural differentiation, and neuronal maturation. All of these aspects are highly relevant to NDDs and neurodevelopment-related psychiatric disorders, for which genetic variations in several NuRD subunits have been identified in the past years. Moreover, the folding of the genomic DNA into higher-order assemblies is increasingly recognized to impact nuclear processes. In this regard, NuRD regulated genes have been recently shown to cluster in 3D space in mouse ESCs, indicating an additional layer in gene regulation beyond the linear chromatin template (Stevens et al., 2017). In any case, the advent of patient-specific iPSC technology offers a promising platform to investigate 2D- and 3D-regulatory effects of genetic variation in NuRD subunits on the development, differentiation, and maturation of living human neurons. Human iPSCs are usually generated from nuclear blood cells or skin fibroblasts through well-established reprogramming methods. In the presence of appropriate signals, human iPSCs can develop and differentiate into nearly any cell type. This applies as well to disease relevant neurons and astroglia, and thus allows to recapitulate, at least in part, altered brain development *in vitro* (Ahmad et al., 2018; Hoffmann et al., 2018).

Interestingly, genetic variation in NuRD subunits and NuRD-regulated genes in NDDs and neurodevelopment-related psychiatric disorders such as SCZ and BD converge on pathways regulating neural development, neuronal maturation, synaptic connectivity and plasticity, and higher cognitive functions. The hypothesis that disturbances taking place in early brain development increase the risk for SCZ has become widely accepted as the “neurodevelopmental hypothesis of SCZ” (Birnbaum and Weinberger, 2017). However, SCZ is still thought to be in terms of nosology, and of pathophysiology and clinical presentation, to be distinct from NDDs. As a case in point, SCZ typically presents in early adulthood, while NDDs such as ASD, ADHD, and ID typically present in childhood. Alternatively, NDDs, including SCZ, may be better conceptualized occupying a continuum in terms of etiology and neurodevelopment. Accordingly, main clinical syndromes appear more likely to reflect the pattern, timing, and severity of perturbed brain development than separate nosology (Owen and O’Donovan, 2017). Such perturbations depend in major part on the severity and aggregated of effects of the underlying genetic lesions. Accordingly, deleterious mutations associate preferentially with the most severe forms of NDDs, while variations encoding more subtle effects associate preferentially with milder forms of NDDs. In any case, genetic variation in NuRD-dependent chromatin remodeling presents an intriguing intersection point in the regulation of neurodevelopment and mental health. Epigenetic mechanisms play a pivotal role in the mediation between the genetic blueprint and the environment that extends from the establishment of gene expression patterns (Jaenisch and Bird, 2003) to experience-dependent adaptations (Hoffmann and Spengler, 2014). Genetic variation in epigenetic mechanisms is thought to modify to varying degrees the response of the genome—for better or for worse (Murgatroyd and Spengler, 2012). Epidemiological studies on SCZ and BD have consistently corroborated the critical role of environmental risk factors, which may explain, at least in part, the current heritability gap (Sullivan and Geschwind, 2019). However, genes–environment interactions appear as well subject to genetic variation. In support of this view, genetic variation in NuRD subunits has been detected in neurodevelopment-related psychiatric disorders. This finding adds to the complexity of genes–environment interactions and may explain why individuals with similar disease burden can present with vastly different outcomes in response to similar environmental exposures.

Epigenetic regulation of gene expression is reversible (Jaenisch and Bird, 2003; Hoffmann and Spengler, 2014) and opens the perspective that NuRD-dependent perturbations in brain development are amenable to therapeutic interventions. Despite such evidence, it will be a challenging task to develop inhibitors that selectively modulate PTMs of NuRD without major off-target effects on unrelated substrates. Therefore, current therapeutic approaches need to address and improve the management of symptoms in particular domains (cognitive, behavioral, sensorimotor) in individuals with perturbed NuRD function. Early brain development presents a time of great vulnerabilities and opportunities. Timely interventions building onto the extraordinary plasticity of the developing brain bear the promise to improve, at least in part, cognitive and behavioral symptoms in NuRD-related disorders and to benefit the affected individuals and their families.

## Author Contributions

AH and DS jointly wrote the manuscript. DS carried out the literature search. AH performed the artwork.

## Funding

Funding for this work was provided by Max Planck Society.

## Conflict of Interest Statement

The authors declare that the research was conducted in the absence of any commercial or financial relationships that could be construed as a potential conflict of interest.
